# Human umbilical cord mesenchymal stem cell‐derived exosome suppresses programmed cell death in traumatic brain injury via PINK1/Parkin‐mediated mitophagy

**DOI:** 10.1111/cns.14159

**Published:** 2023-03-08

**Authors:** Li Zhang, Yixing Lin, Wanshan Bai, Lean Sun, Mi Tian

**Affiliations:** ^1^ Department of Neurosurgery, Jinling Hospital, School of Medicine Nanjing University Nanjing China; ^2^ Department of Anesthesiology Affiliated Zhongda Hospital of Southeast University Nanjing China

**Keywords:** exosome, ferroptosis, human umbilical cord mesenchymal stem cell, mitophagy, neuroprotection, pyroptosis, traumatic brain injury

## Abstract

**Aims:**

Recently, human umbilical cord mesenchymal stem cell (HucMSC)‐derived exosome is a new focus of research in neurological diseases. The present study was aimed to investigate the protective effects of HucMSC‐derived exosome in both in vivo and in vitro TBI models.

**Methods:**

We established both mouse and neuron TBI models in our study. After treatment with HucMSC‐derived exosome, the neuroprotection of exosome was investigated by the neurologic severity score (NSS), grip test score, neurological score, brain water content, and cortical lesion volume. Moreover, we determined the biochemical and morphological changes associated with apoptosis, pyroptosis, and ferroptosis after TBI.

**Results:**

We revealed that treatment of exosome could improve neurological function, decrease cerebral edema, and attenuate brain lesion after TBI. Furthermore, administration of exosome suppressed TBI‐induced cell death, apoptosis, pyroptosis, and ferroptosis. In addition, exosome‐activated phosphatase and tensin homolog‐induced putative kinase protein 1/Parkinson protein 2 E3 ubiquitin–protein ligase (PINK1/Parkin) pathway‐mediated mitophagy after TBI. However, the neuroprotection of exosome was attenuated when mitophagy was inhibited, and PINK1 was knockdown. Importantly, exosome treatment also decreased neuron cell death, suppressed apoptosis, pyroptosis, and ferroptosis and activated the PINK1/Parkin pathway‐mediated mitophagy after TBI in vitro.

**Conclusion:**

Our results provided the first evidence that exosome treatment played a key role in neuroprotection after TBI through the PINK1/Parkin pathway‐mediated mitophagy.

## Graphical abstract

HucMSC‐derived exosome suppressed apoptosis, pyroptosis, and ferroptosis through the PINK1/Parkin pathway‐mediated mitophagy after TBI, resulting in decreased brain damage.

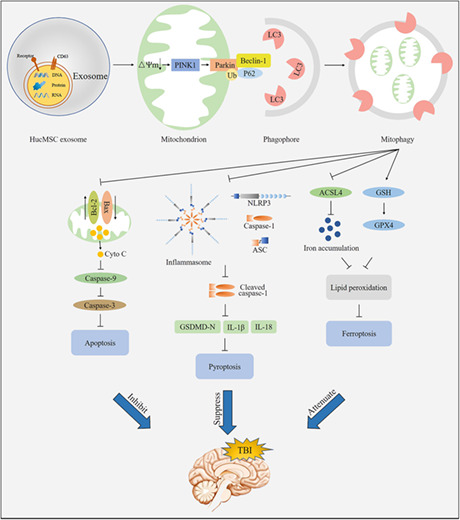

## 1 INTRODUCTION

Traumatic brain injury (TBI) occurs due to direct impact or hit on the head caused by factors such as motor vehicles, crushes, and assaults.^1^ Central nervous system (CNS) is highly sensitive to external mechanical damage, presenting a limited capacity for regeneration due to its inability to restore either damaged neurons or synaptic network.^2^ Thus, TBI is identified as an important global health concern that represents a leading cause of death and disability.^3^ The primary injury happens at the time of injury and is responsible for the initiation of secondary injury cascades such as inflammation, apoptosis, oxidative stress, and endoplasmic reticulum.^4^ These cascades contribute to long‐term brain damage including neurological deficits, brain edema, and blood brain barrier (BBB) disruption.^5^ Despite the progress has been made in the prevention and treatment of TBI in the past, patients suffering from TBI usually end up with poor prognosis.^6^ Thus, it is urgently needed to find optimal therapies and improve patients' long‐term neurological functioning after TBI.

Recently, cell therapies, especially human umbilical cord mesenchymal stem cell (HucMSC) transplantation has been suggested to be a powerful method for the treatment of TBI.^7^ HucMSC exhibits strong proliferative ability, low immunogenicity, and multi‐potential differentiation.^8^ Exogenous HucMSC can stimulate repair processes, suppress astrocyte activation, inhibit inflammatory factors, and decrease neuron loss after brain injury, resulting in improvement of behavioral outcomes and cerebral lesion volumes after brain damage.^9^ It has been found that the therapeutic effects of HucMSC were depend on the release of exosome. Exosome is cup‐like shapes with a diameter size range from 30 to 150 nm, it is produced by the membrane of multivesicular body (MVB).^10^ When the endosome or MVB fuse with the plasma membrane, exosome is released extracellularly.^11^ Exosome contains lipids, proteins, non‐coding RNAs, and mRNAs, these contents are protected from degradation by the lipid bilayer of exosome.^12^ Via releasing these components to neighboring cells, exosome is able to regulate cell‐to‐cell communications as well as multiple autocrine and paracrine cellular phenotypes.^13^ In addition, exosome has extensive and unique advantages due to its favorable pharmacokinetic and ability to penetrate physiological barriers.^14^ Exosome readily crosses the BBB and has the potential to specifically deliver molecules to CNS.^11^ Recently, studies have revealed the effective role of exosome in the diagnosis and treatments of CNS diseases such as TBI.^15^

It has been shown that HucMSC could provide neuroprotection after TBI.^16^ However, the role of HucMSC‐derived exosome in TBI has not been fully studied. In this study, we explored whether HucMSC‐derived exosome could suppress cell death, apoptosis, pyroptosis, and ferroptosis after TBI and the underlying mechanisms.

## 2 MATERIALS AND METHODS

### 2.1 Animal preparation

All animal studies were carried out in accordance with the principles of the ARRIVE (Animal Research: Reporting of In Vivo Experiments) guidelines and were approved by the Institutional Animal Care and Use Committee of Nanjing University (Nanjing, China). Male ICR mice (28–32 g) were obtained from Animal Center of Jinling hospital. Mice were housed on a 12 h light/dark cycle at 23 ± 1°C with free access to food and water.

### 2.2 Primary culture of mouse cortical neurons

The culture of mouse cortical neurons was performed according to previous studies.^17^, ^18^ In brief, mouse cortical neurons were isolated from the embryos of time‐mated pregnant mice and subsequently cultured in poly‐D‐lysine‐coated 6‐well dishes at a density of 1 × 10^6^ cells per well. Then, the neurons were cultured in neurobasal medium (Life Technologies, USA, catalog number: 21103049) supplemented with 2% B27 (Life Technologies, USA, catalog number: 17504044) and 1 mM glutamate (Sigma Aldrich, USA, catalog number: G3291) at 37°C on 5% CO_2_ incubator. Half of the culture medium was replaced with fresh medium every 3 days. The in vitro studies were performed after culture of 10–12 days.

### 2.3 TBI models

The in vivo (mouse) TBI model was performed using a Controlled Cortical Impact (CCI) model. Mice were anesthetized with 3% isoflurane and subsequently placed on the stereotaxic apparatus. With a 1.5 cm midline longitudinal incision of the scalp, the skull was exposed. The area to be impacted lies on the right frontal skull (2.5 mm lateral to the midline and 0.5 mm anterior to bregma). After confirming the correct impact location again, mice were subjected to cortical contusion injury by a 3.0 mm rounded impactor tip (piston velocity: 3.5 m/s; deformation depth: 1.5 mm, dwell time: 150 ms). The mortality results from apnea were decreased by early respiratory support. Then the mice were returned to cages to recover for 24 h with free access to a standard diet. The sham‐injured mice underwent the same procedures but did not undergo the CCI.

The in vitro (neurons) TBI model was conducted using a mechanical stretch injury model. Briefly, the 6‐well plates were manually scratched with a sterile plastic needle followed by a 9 × 9 square grid (the space between every line was 4 mm). Then, neurons were cultured at 37°C in 5% CO_2_ incubator for another 24 h without change of culture medium.

### 2.4 Exosome isolation and characterization

Extraction of HucMSCs‐derived exosome: after expanded culture, HucMSCs grew to about 85%, the culture medium was discarded, the cells were washed three times with sterile phosphate buffer saline (PBS), and serum‐free culture medium was added. After 48 h of culture, the cell supernatant was collected and transferred to a 50 mL centrifuge tube. After centrifugation at 500 *g*, 4°C for 5 min, the supernatant was collected and then transferred to a new 50 mL centrifuge tube, centrifugated at 2000 *g*, 4°C for 30 min. Then, the supernatant was collected and centrifugated at 10,000 *g* for 60 min. Finally, the supernatant was collected, filtered with 0.22 μM sterile filter, and added to the ultra‐high speed centrifugal tube. After centrifugation at 120,000 *g*, 4°C for 1 h, the supernatant was removed, and the precipitate was dissolved with 200 μL sterile PBS and resuspended.

Measurement of the exosome particle size: exosomes were diluted 40 times with sterile PBS and then filtered with 0.22 μM sterile filter. The particle size of exosomes was detected by nanoparticle tracking analysis (NTA) at Vivacell Biosciences with ZetaView PMX 110 (Particle Metrix, Meerbusch, Germany) as previously described.^19^

Detection of exosome by transmission electron microscopy (TEM): exosomes were washed with 1 mL of PBS for three times. Then, 0.5 mL of 2% osmic acid solution was added and fixed at 4°C for 2 h. After washing with 1 mL of PBS for three times, 1 mL of 50%, 70%, 80%, and 90% ethanol were used to gradually dehydrate for 15 min each time and then 1 mL of 100% ethanol was used to dehydrate twice for 20 min each time. After dehydration, the sample was replaced twice with 1 mL acetone for 15 min each time. Then, the sample was immersed and put into the embedding plate, and the embedding plate was polymerized at 65°C for 48 h. Finally, it was stained with uranyl acetate and lead acetate for 10 min. After cleaning, exosomes were observed using TEM (JEM‐1230, JEOL Ltd., Akishima, Japan) to identify the morphology.

### 2.5 Experimental design

**FIGURE 1:**
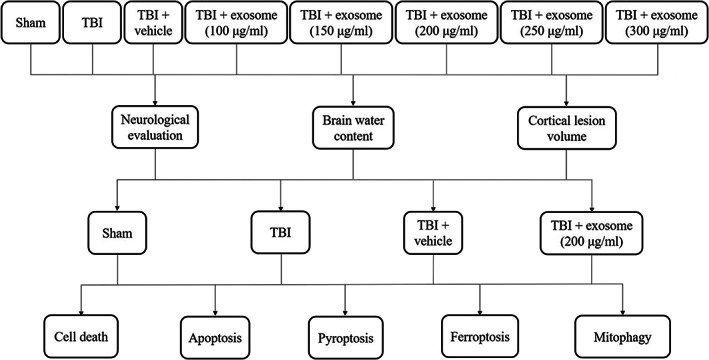
Diagram showing the experimental design throughout the study.

The design of our experiment was shown in Figure 1. For in vivo studies, mice were randomly divided into eight groups as follows: sham, TBI, TBI + vehicle, TBI + exosome (five different dose groups: 100 μg/mL, 150 μg/mL, 200 μg/mL, 250 μg/mL, 300 μg/mL). Either exosome or an equal volume of vehicle (PBS) was administrated 30 min after TBI by tail vein injection.

For in vitro experiments, neurons were divided into six groups as follows: control, TBI, TBI + PBS, and TBI + exosome (three different dose groups: 50 μg/mL, 100 μg/mL, 150 μg/mL). Exosome was first dissolved in PBS and then added to cultured media to reach different final concentrations. The concentrations of exosome used in vivo and in vitro studies were according to our preliminary experiments.

### 2.6 Neurological evaluation and brain water content

The neurological impairment was evaluated using the neurologic severity score (NSS) at 1 day, 3 days and 7 days after TBI. The investigators estimate the ability of mouse to perform 10 different tasks which demonstrate physiological behavior, alertness, and motor function. One point is given for failing to perform each task, thus 0 = minimum deficit and 10 = maximum deficit (Table 1).^20^, ^21^

**TABLE 1:** Neurological severity scoring (NSS).

Items	Description	Points	
		Success/failure	
Exit circle	Ability and initiative to exit a circle of 30 cm diameter (time limit: 3 min)	0	1
Mono−/hemiparesis	Paresis of upper and/or lower limb of contralateral side	0	1
Straight walk	Alertness, initiative, and motor ability to walk straight, when placed on the floor	0	1
Startle reflex	Innate reflex (flinching in response to a loud hand clap)	0	1
Seeking behavior	Physiological behavior as a sign of “interest” in the environment	0	1
Beam balancing	Ability to balance on a beam 7 mm in width for at least 10 s	0	1
Round stick balancing	Ability to balance on a round stick 5 mm in diameter for at least 10 s	0	1
Beam walk: 3 cm	Ability to cross a beam (length × width, 30 × 3 cm)	0	1
Beam walk: 2 cm	Same task but with increased difficulty (beam width = 2 cm)	0	1
Beam walk: 1 cm	Same task but with increased difficulty (beam width = 1 cm)	0	1
Maximum score			10

The motor performance of mice was also evaluated at 1 day, 3 days, and 7 days after TBI using the grip test score^18^ and neurological score.^22^ Both tests were carried out by an investigator who was blinded to the experimental groups. For grip test score, mice were placed on a thin, horizontal, metal 45 cm long wire which was lay up between two vertical poles 45 cm above a foam pad. Zero point was given if the mice was unable to remain on the wire for <30 s; one point was given if the mice failed to hold on to the wire with both hind paws and forepaws together; two points were given if the mice held on to the wire with both hind paws and forepaws but not the tail; three points were given if the mice used its tail along with both hind paws and forepaws; four points were given if the mice moved along the wire on all four paws plus tail; five points were given if mice that scored four points also ambulated down one of the posts used to support the wire. The grip test was performed in tree times and a total point was calculated for each mouse. For neurological score, an 18‐point scoring system, ranging from 3 to 18, assessed the neurological deficits from six subtests. These tests comprise spontaneous activity (0–3 score), symmetry in all limbs' movements (0–3 score), forelimb extension (0–3 score), climbing (1–3 score), body proprioception (1–3 score), and response to vibrissae stimulation (1–3 score). Higher scores indicate better neurological function.

The brain water content was conducted according to a previous study.^21^ Briefly, mouse brain was taken out and placed onto a cooled brain matrix 1 day following TBI. The cerebellum and stem were taken away, and the ipsilateral tissue was weighed to get the wet weight (ww). Then, the hemisphere was dried for 72 h at 80°C and weighed again to get the dry weight (dw). The brain water content equals (ww−dw)/ww × 100%.

### 2.7 Measurement of lesion volume

Lesion volume measurement was conducted based on previous studies.^18^ Briefly, the brain sections were stained with cresyl violet. Then, the areas of lesion, injured, and non‐injured brain tissues were estimated by an image analysis system. Area measurements from each section were obtained and summed, and corresponding volumes were calculated.

### 2.8 Nissl staining, terminal deoxynucleotidyl transferase‐mediated dUTP nick 3′‐end labeling (TUNEL) staining and propidium iodide (PI) staining

Both Nissl staining and TUNEL staining were conducted according to our previous study.^23^ For Nissl staining, the 4 μm sections were treated with Nissl staining solution (Beyotime Biotechnology, Shanghai, China; catalog number: C0117) for 5 min. Neurons in five randomly selected areas were counted under the microscope by experimenters who had no prior known the group assignments. For TUNEL staining, the sections were incubated with TUNEL staining solution (Beyotime Biotechnology, Shanghai, China; catalog number: C1088) for 1 h. The brain injury was expressed by the apoptotic index. For PI staining, the sections were incubated with PI staining solution (Beyotime Biotechnology, Shanghai, China; catalog number: ST512) for 20 min. Subsequently, the stained sections were observed under a fluorescence microscope. The apoptotic‐positive cells and PI‐positive cells were measured by two independent observers blinded to the groups.

### 2.9 Isolation of mitochondria

We used a tissue mitochondria isolation kit (Beyotime Biotechnology, Shanghai, China; catalog number: C3606) to separate cytoplasmic and mitochondria fractions. The mitochondria fractions separated from tissues were further lysed for extraction of mitochondrial proteins.

### 2.10 Western blot analysis

Western blot analysis was performed according to previous studies.^24^ Proteins were separated by 8–12% sodium dodecyl sulfate‐polyacrylamide gel electrophoresis (SDS‐PAGE), transferred to polyvinylidene fluoride (PVDF) membranes and incubated overnight at 4°C with the following primary antibodies: CD9 (1:1000; Abcam, Cambridge, MA, USA; catalog number: ab236630), CD63 (1:1000; Abcam, Cambridge, MA, USA; catalog number: ab217345), B‐cell lymphoma 2 (Bcl‐2) (1:1000; Cell Signaling Technology, Danvers, MA, USA; catalog number: 3498), Bcl‐2‐associated X protein (Bax) (1:1000; Cell Signaling Technology, Danvers, MA, USA; catalog number: 14796), cytochrome c (1:1000; Abcam, Cambridge, MA, USA; catalog number: ab133504), voltage‐dependent anion channel 1 (VDAC1) (1:1000; Abcam, Cambridge, MA, USA; catalog number: ab133504), cleaved caspase‐3 (1:1000; Cell Signaling Technology, Danvers, MA, USA; catalog number: 9661), cleaved caspase‐1 (1:1000; Cell Signaling Technology, Danvers, MA, USA; catalog number: 89332), N‐terminal domains of gasdermin D (GSDMD‐N) (1:1000; Cell Signaling Technology, Danvers, MA, USA; catalog number: 10137), NLR family pyrin domain containing 3 (NLRP3) (1:1000; Cell Signaling Technology, Danvers, MA, USA; catalog number: 15101), transferring receptor (TfR) (1:1000; Abcam, Cambridge, MA, USA; catalog number: ab84036), ferritin (Ft) (1:1000; Abcam, Cambridge, MA, USA; catalog number: ab75973), ferroportin‐1 (Fpn‐1) (1:1000; Abcam, Cambridge, MA, USA; catalog number: ab58695), acyl coenzyme A synthetase long‐chain family member 4 (ACSL4) (1:1000; Abcam, Cambridge, MA, USA; catalog number: ab155282), glutathione peroxidase 4 (GPX4) (1:1000; Abcam, Cambridge, MA, USA; catalog number: ab125066), translocase of outer mitochondrial membrane 20 (TOMM20) (1:1000; Cell Signaling Technology, Danvers, MA, USA; catalog number: 42406), cytochrome c oxidase IV (COX IV) (1:1000; Cell Signaling Technology, Danvers, MA, USA; catalog number: 11967), Beclin‐1 (1:1000; Novus Biological, Littleton, CO, USA; catalog number: NB500‐249), microtubule‐associated protein light chain 3 (LC3) (1:1000; Novus Biological, Littleton, CO, USA; catalog number: NB600‐1384), phosphatase and tensin homolog induced kinase 1 (PINK1) (1:1000; Abcam, Cambridge, MA, USA; catalog number: ab23707), Parkinson protein 2 E3 ubiquitin‐protein ligase (Parkin) (1:1000; Abcam, Cambridge, MA, USA; catalog number: ab77924), and β‐actin (1:5000; Bioworld Technology, Minneapolis, MN, exosome; catalog number: AP0060). Subsequently, the membranes were incubated with corresponding secondary antibodies for 2 h at room temperature. The protein bands were visualized by western blot detection reagents (Millipore‐Sigma, Burlington, MA, USA). The ImageJ software quantified band intensities. All original blot images were available in Appendix S1.

### 2.11 Immunofluorescence (IF) staining

Immunofluorescence staining was assessed according to a previous immunostaining protocol as follows: the slides of each coronal section were incubated in blocking buffer for 2 h, and then washed three times with PBS for 10 min. Afterward, sections were incubated with specific primary antibodies for cleaved caspase‐1 (1:100; Cell Signaling Technology, Danvers, MA, USA; catalog number: 89332), GSDMD‐N (1:100; Cell Signaling Technology, Danvers, MA, USA; catalog number: 10137), NLRP3 (1:100; Cell Signaling Technology, Danvers, MA, USA; catalog number: 15101), 8‐hydroxyguanosine (8‐OHdG) (1:100; Bioss, Massachusetts, Boston, USA; catalog number: bs‐1278R), ACSL4 (1:200; Abcam, Cambridge, MA, USA; catalog number: ab155282), GPX4 (1:200; Abcam, Cambridge, MA, USA; catalog number: ab125066), and LC3 (1:200; Novus Biological, Littleton, CO, USA; catalog number: NB600‐1384), the slides kept overnight in a dark place at 4°C. Subsequently, after washed three times with PBS, the slides were incubated with anti‐NeuN antibody (1:100; Cell Signaling Technology, Danvers, MA, USA; catalog number: 24307) or anti‐Iba‐1 antibody (1:100; Cell Signaling Technology, Danvers, MA, USA; catalog number: 17198) under similar conditions. The following day, thoroughly washed three times with PBS, the slides were incubated with the corresponding secondary antibodies for 1 h at room temperature. After washing three times with PBS, the slides were stained with 2‐(4‐amidinophenyl)‐6‐indolecarbamidine dihydrochloride (DAPI; Beyotime Biotechnology, Shanghai, China; catalog number: C1002) for 2 min to show the locations of nuclei. Then, coverslips were used with the fluorescence quenching agent. We imaged the fluorescently stained cells via Olympus IX71 inverted microscope system and analyzed using Image‐Pro Plus 6.0 software (Media Cybernetics, Silver Spring, MD).

### 2.12 ELISA

Brain samples and culture medium were collected. The levels of interleukin (IL)‐1β, IL‐6, IL‐18, and tumor necrosis factor‐α (TNF‐α) were measured with IL‐1β (Beyotime Biotechnology, Shanghai, China; catalog number: PI301), IL‐6 (catalog number: PI326), IL‐18 (catalog number: PI553), and TNF‐α (catalog number: PT512) ELISA kits according to the manufacturer instructions.

### 2.13 Measurement of malondialdehyde (MDA) and glutathione peroxidase (GPx)

The injured cerebral cortex samples were homogenized in 2 mL of PBS. After centrifugation at 10,000 *g* for 25 min, the MDA and GPx in the supernatant were measured using an MDA assay kit (Beyotime Biotechnology, Shanghai, China; catalog number: S0131M) and GPx assay kit (Beyotime Biotechnology, Shanghai, China; catalog number: S0056), and detected by a spectrophotometer according to the manufacturer's instructions.

### 2.14 Perls' diaminobenzidine (DAB) staining

The injured cerebral cortex samples were fixed with 4% paraformaldehyde for 48 h and embedded in paraffin. Samples were cut into 4 μm thick slices for using a Perls' DAB stain kit (ChemicalBook, Beijing, China; catalog number: CB25533488) according to the manufacturer's instructions. In short, after dewaxing, staining, and color development, the sections were mounted in glycerinum. Finally, six pictures (400×) from each section were taken and quantitation using Image‐Pro Plus system.

### 2.15 Observation of mitochondria by TEM

Transmission electron microscopy was used to identify mitochondria as previously described.^25^ Briefly, at 1 day after TBI, mice were killed and perfused with 2.5% buffered glutaraldehyde. The specimens were collected and fixed in glutaraldehyde with a 1% (w/v) solution of osmium tetroxide. The fixed specimens were then embedded, sectioned, double stained with lead citrate, and uranyl acetate, observed under a TEM JEM‐1011 (JEOL, Japan).

### 2.16 Cell viability analysis

To analyze the cell viability of primary cultured neuron cells, we first used the trypan blue (TB) staining assay. Cells were stained by 0.4% TB (Beyotime Biotechnology, Shanghai, China; catalog number: ST798) after treatment. Stained cells were considered as dead while unstained cells were considered as viable. The number of TB‐positive cells and total cell number were counted. Survival value equals = (number of stained cells/number of total cells) × 100%.

In addition, a lactate dehydrogenase (LDH) cytotoxicity assay kit (Beyotime Biotechnology, Shanghai, China; catalog number: C0016) was used to confirm the results of TB staining. In brief, cells were treated with LDH release agent, and the culture medium was centrifuged. The supernatant was further collected to evaluate the activity of LDH. The OD value at 490 nm was analyzed by a spectrophotometer. The percentage of damaged cells (%) = (OD490_exosomemple_–OD490_media_)/(OD490_maximum_–OD490_media_) × 100%. OD490_maximum_ = cells treated with LDH release agent and OD490_media_ = only media without any cells.

### 2.17 Measurement of neuron lipid peroxides

Lipid peroxides in neuron were measured by C11‐BODIPY 581/591 (Amgicam, Wuhan, China; catalog number: ajci64572) staining. Briefly, cells were stained with BODIPY (5 μM) for 30 min at 37°C for 30 min in the dark. Then, cells were washed with PBS three times and observed immediately under a fluorescent microscopy (Carl Ziess, Germany). The fluorescence images were collected using a single rapid scan. In addition, we analyzed the levels of lipid peroxides by a fluorescence microplate reader (BioTek, USA). Cells were stained with BODIPY, washed with PBS and centrifugated for 5 min at 1000 × *g*, the supernatant was removed, and the remaining cells were resolved with 1% Triton X‐100. Fluorescence was analyzed at an excitation wavelength of 500 nm and an emission wavelength of 510 nm.

### 2.18 Statistical analysis

All statistical analysis was performed with SPSS 19.0 (SPSS Inc., Chicago, IL). The Shapiro–Wilk test was used to confirm whether the data fit a normal distribution. Data were summarized as mean ± SD (normally distributed) or median (non‐normally distributed). For normally distributed data, one‐way analysis of variance (ANOVA) followed by Tukey's test was used for multiple group comparisons. The least significant difference (LSD) post hoc test was used to determine statistical differences between groups. Statistical differences between the two groups were compared using Student's *t*‐test. For data that did not conform to a normal distribution, comparisons between groups were made using the Mann–Whitney *U*‐test. A value of *p* < 0.05 was considered statistically significant.

## 3 RESULTS

### 3.1 HucMSC‐derived exosome provided neuroprotection after TBI

**FIGURE 2:**
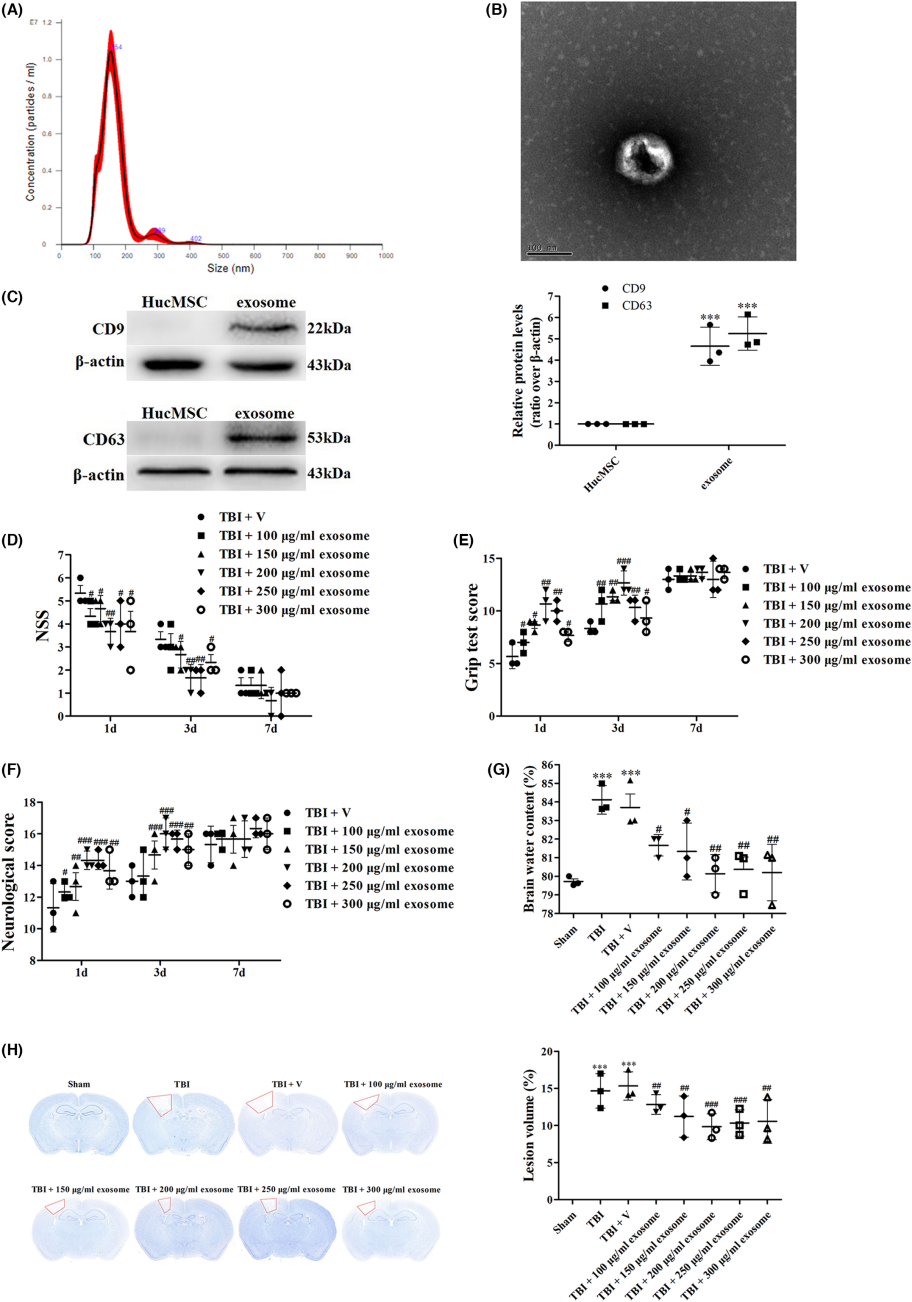
Characteristics and effects of HucMSC‐derived exosome in TBI. (A) The size of exosome was assessed by NTA. (B) Exosome were observed under TEM. Scale bar: 100 Nm. (C) Western blot assay for the expression of exosome markers CD9 and CD63. (D, E, F) Mice were subjected to TBI and received 100 μg/mL, 150 μg/mL, 200 μg/mL, 250 μg/mL, and 300 μg/mL of exosome or vehicle 30 min after TBI. NSS (D), grip test score (E), and neurological score (F) were evaluated at 1, 3, and 7 days after TBI and exosome treatment. (G, H) Mice were subjected to TBI and received 100 μg/mL, 150 μg/mL, 200 μg/mL, 250 μg/mL, and 300 μg/mL of exosome or vehicle 30 min after TBI. Brain water content (G) and brain tissue loss (H) were examined at 1 day after TBI and exosome treatment. Data were presented as mean ± SD; ***p < 0.001 versus sham group; ^#^ *p* < 0.05, ^##^ *p* < 0.01, ^###^ *p* < 0.001 versus TBI + vehicle group. β‐Actin was used as a loading control.

To determine whether HucMSC‐derived exosome (short for exosome) protected mice against TBI, exosome was isolated from HucMSC as described in Methods. The particle size of exosome was analyzed by NTA. We found that the average particle size of exosome was 146.8 nm, and the main peak of particle size was 153.2 nm (Figure 2A). Moreover, the shape of exosome was observed by TEM (150,000×) (Figure 2B). In addition, we detected the expression of the exosome surface marker CD9 and CD63 by Western blot, the results showed that the expression of CD9 and CD63 was significantly higher than that of HucMSC (Figure 2C).

We then examined the protective effects of exosome in TBI. We firstly used the NSS to evaluate the neurological impairment of mice after TBI. As showed in Figure 2D, exosome‐treated mice showed better neurological function than vehicle‐treated mice at 1 day. Moreover, a significant difference was still detectable at 3 days. However, there was no obvious difference between these two groups at 7 days (*p* > 0.05).

In addition, we used grip test score and neurological score to measure the motor performance of mice following TBI. Figure 2E,F indicated that the motor performance of exosome‐treated mice was obviously better than that of the vehicle‐treated mice at 1 day and 3 days. However, there was no significant difference between these two groups at 7 days (*p* > 0.05).

Next, we used brain water content to validate the neuroprotective effects of exosome. As shown in Figure 2G, the brain edema was increased at 1 day after TBI. However, treatment of exosome decreased the brain edema. Significantly, all of NSS, grip test score, neurological score, and brain water content experiments suggested that 200 μg/mL of exosome showed the best neuroprotection.

Finally, we explored whether exosome could affect TBI‐induced cortical lesion volume. Figure 2H showed that TBI induced significant brain tissue loss (red lines). While exosome treatment decreased the lesion volume (Figure 2H). In conclusion, all experiments above indicated that exosome could provide neuroprotection after TBI and 200 μg/mL of exosome exhibited the best neuroprotection, which we would use in our subsequent in vivo experiments.

### 3.2 Exosome reduced cell death and apoptosis after TBI

**FIGURE 3:**
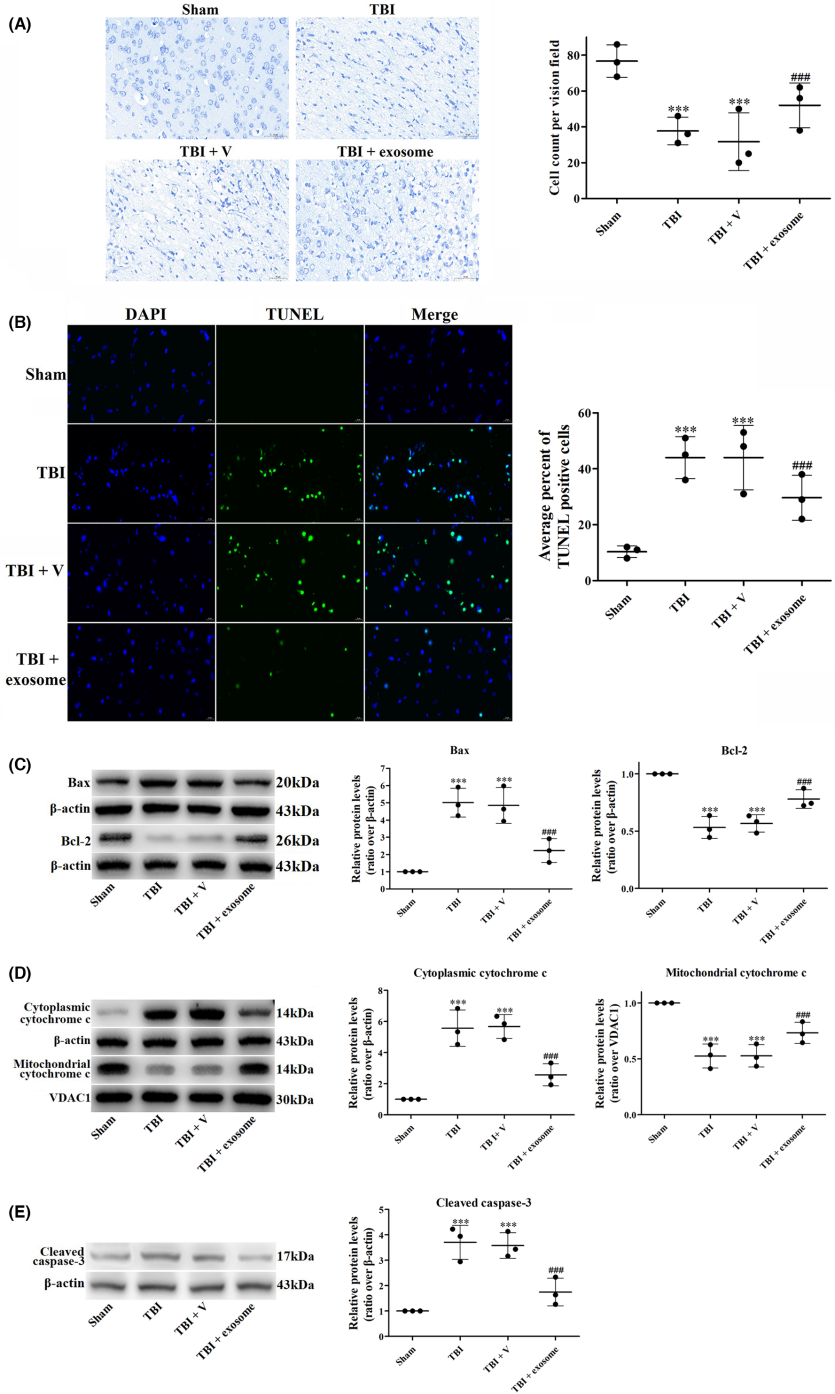
Exosome suppressed TBI‐induced neuron cell death and apoptosis. (A) Nissl staining was used to visualize the neuronal cell outline and structure in all groups. (B) TUNEL staining was used to examine the percentage of apoptotic cells in all groups. (C) Western blot assay for the expression of Bax and Bcl‐2 in the ipsilateral cortex after TBI and exosome treatment. (D) Western blot assay for the expression of cytoplasmic and mitochondria cytochrome c in the ipsilateral cortex after TBI and exosome treatment. (E) Western blot assay for the expression of cleaved caspase‐3 in the ipsilateral cortex after TBI and exosome treatment. Data were presented as mean ± SD; ***p < 0.001 versus sham group; ^###^ *p* < 0.001 versus TBI + vehicle group. Scale bar of Nissl staining: 50 μm. Scale bar of TUNEL staining: 20 μm. VDAC1 Was used as a loading control for mitochondria extracts. β‐Actin was used as a loading control for cytoplasmic and whole‐cell extracts.

In the pathophysiology of TBI, cell death runs through the occurrence and development. Moreover, apoptosis plays an essential part in the progress of TBI, and its inhibition may help overcome TBI's negative consequences and improve functional recovery.^26^ To study the occurrence of neural cell death, we used Nissl staining. In the sham group, the neurons were intact and clear, without edema around the cells (Figure 3A). While in the TBI and TBI + vehicle groups, the damaged neuron cells were increased, exhibiting extensive degenerative changes including swollen cell bodies, shrunken cytoplasm, and oval or triangular nucleus (Figure 3A). On the contrary, the severity of neuron damage was remarkably attenuated after exosome treatment (Figure 3A).

To examine TBI‐induced neural cell apoptosis, we applied TUNEL fluorescence staining. As shown in Figure 3B, few apoptotic‐positive cells were detected in the sham group. However, apoptotic‐positive cells were found in the TBI and TBI + vehicle groups (Figure 3B). While treatment of exosome significantly decreased the number of apoptotic‐positive cells (Figure 3B).

To further confirm the effects of exosome on apoptosis, we analyzed several apoptosis markers such as Bax, Bcl‐2, cytochrome c, and caspase‐3. The results showed that the expression of Bcl‐2 and mitochondrial cytochrome c was down‐regulated while the expression of Bax, cytoplasmic cytochrome c, and cleaved caspase‐3 was up‐regulated after TBI (Figure 3C–E). Treatment of exosome suppressed TBI‐induced apoptosis compared with the vehicle‐treated group (Figure 3C–E).

### 3.3 Exosome suppressed pyroptosis after TBI

**FIGURE 4:**
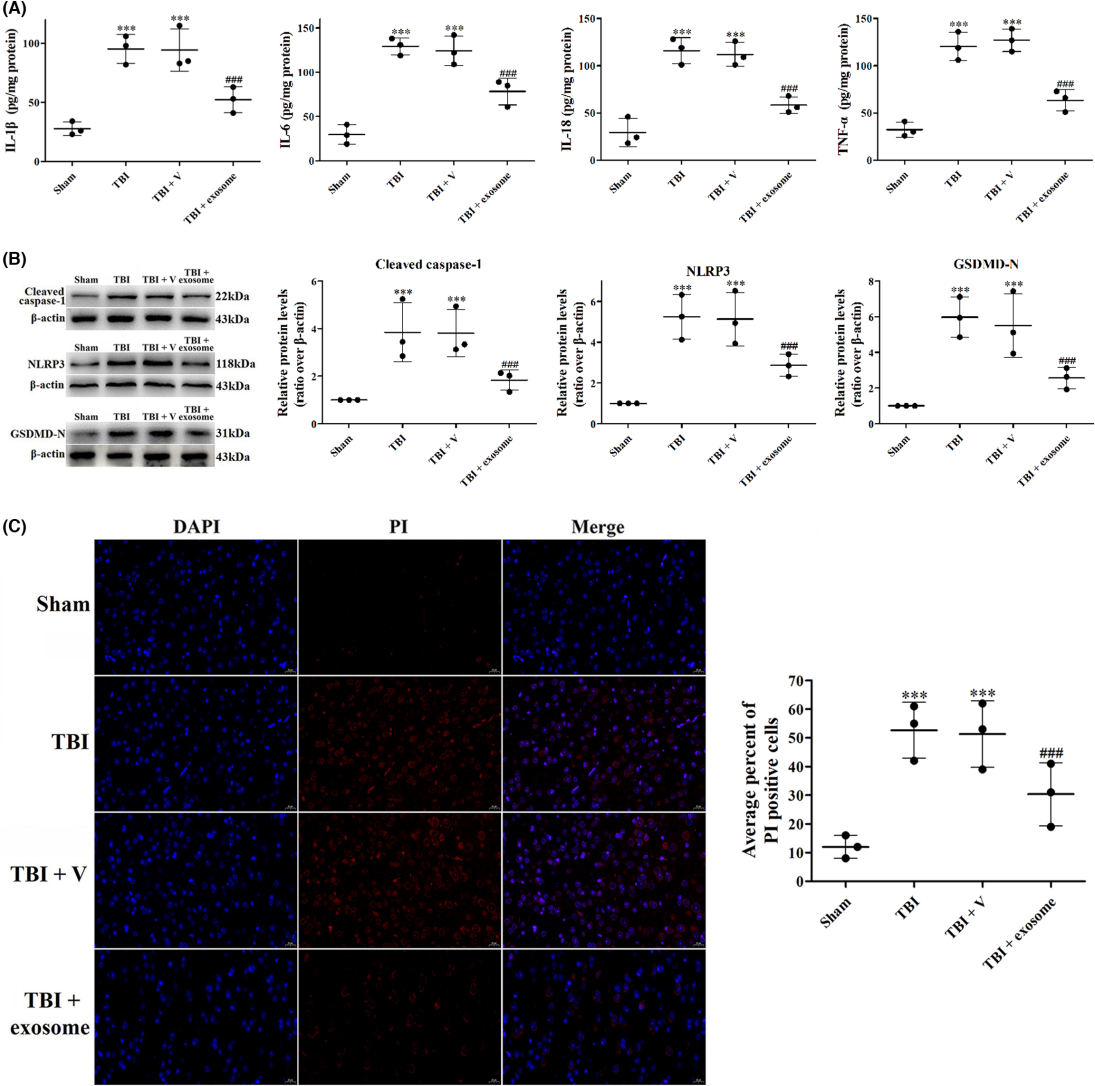
Exosome suppressed TBI‐induced pyroptosis. (A) Effects of exosome on inflammatory cytokines after TBI and exosome treatment. Quantification of IL‐1β, IL‐6, IL‐18, and TNF‐α levels in all groups. (B) Western blot assay for the expression of cleaved caspase‐1, NLRP3, and GSDMD‐N in the ipsilateral cortex after TBI and exosome treatment. (C) PI staining was used to examine the percentage of inflammatory necrosis cells in all groups. Data were presented as mean ± SD; ***p < 0.001 versus sham group; ^###^ *p* < 0.001 versus TBI + vehicle group. Scale bar: 20 μm. β‐Actin was used as a loading control.

It has been shown that pyroptosis was an important cause of neuronal damage after TBI. Pyroptosis is a type of inflammatory programmed cell death (PCD) that is characterized by the release of pro‐inflammatory cytokines such as interleukin (IL), which triggers an inflammatory cascade response that results in cellular damage.^27^ Therefore, we first measured the levels of pro‐inflammation cytokines including IL‐1β, IL‐6, IL‐18, and TNF‐α by ELISA. We found that the levels of IL‐1β, IL‐6, IL‐18 and TNF‐α were increased at 1 day after TBI, which were significantly inhibited by exosome treatment compared to vehicle‐treated group (Figure 4A), indicating that exosome could inhibit TBI‐induced inflammation.

We then examined some pyroptosis‐related markers, including the expression of cleaved caspase‐1, NLRP3, GSDMD‐N, and PI staining. After TBI, the expression of cleaved caspase‐1, NLRP3, GSDMD‐N (Figure 4B) and the percentage of PI‐positive cells (Figure 4C) were increased. In contrast, exosome treatment partly abolished these effects of TBI (Figure 4B,C), suggesting that exosome could attenuate TBI‐induced pyroptosis.

**FIGURE 5:**
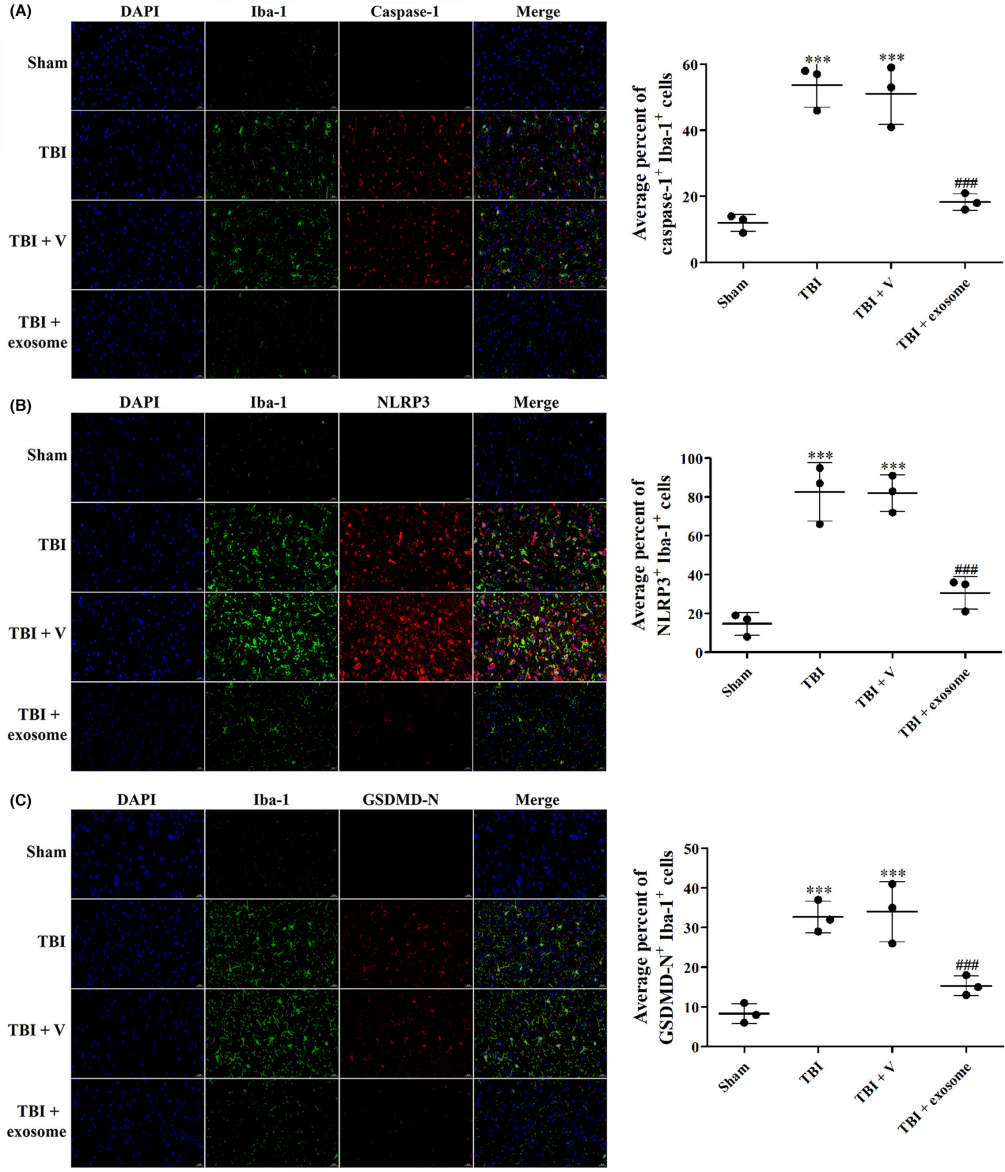
Exosome decreased the level of pyroptosis‐related proteins. (A–C) Effects of exosome on pyroptosis‐related proteins at 1 day after TBI and exosome treatment. Representative images of IF staining for cleaved caspase‐1 and Iba‐1 (A), NLRP3, and Iba‐1 (B), GSDMD‐N and Iba‐1 (C). Data were presented as mean ± SD; ***p < 0.001 versus sham group; ^###^ *p* < 0.001 versus TBI + vehicle group. Scale bar: 20 μm.

To better understand the anti‐pyroptosis effect of exosome, we performed double immunofluorescence staining of Iba‐1 and three important components of pyroptosis: NLRP3, GSDMD‐N and cleaved caspase‐1. As shown in Figure 5, the number of both cleaved caspase‐1^+^ and Iba‐1^+^ (Figure 5A), NLRP3^+^ and Iba‐1^+^ (Figure 5B), GSDMD‐N^+^ and Iba‐1^+^ (Figure 5C) cells in the brain cortex of TBI mice were significantly reduced after exosome treatment.

### 3.4 Exosome decreased ferroptosis after TBI

**FIGURE 6:**
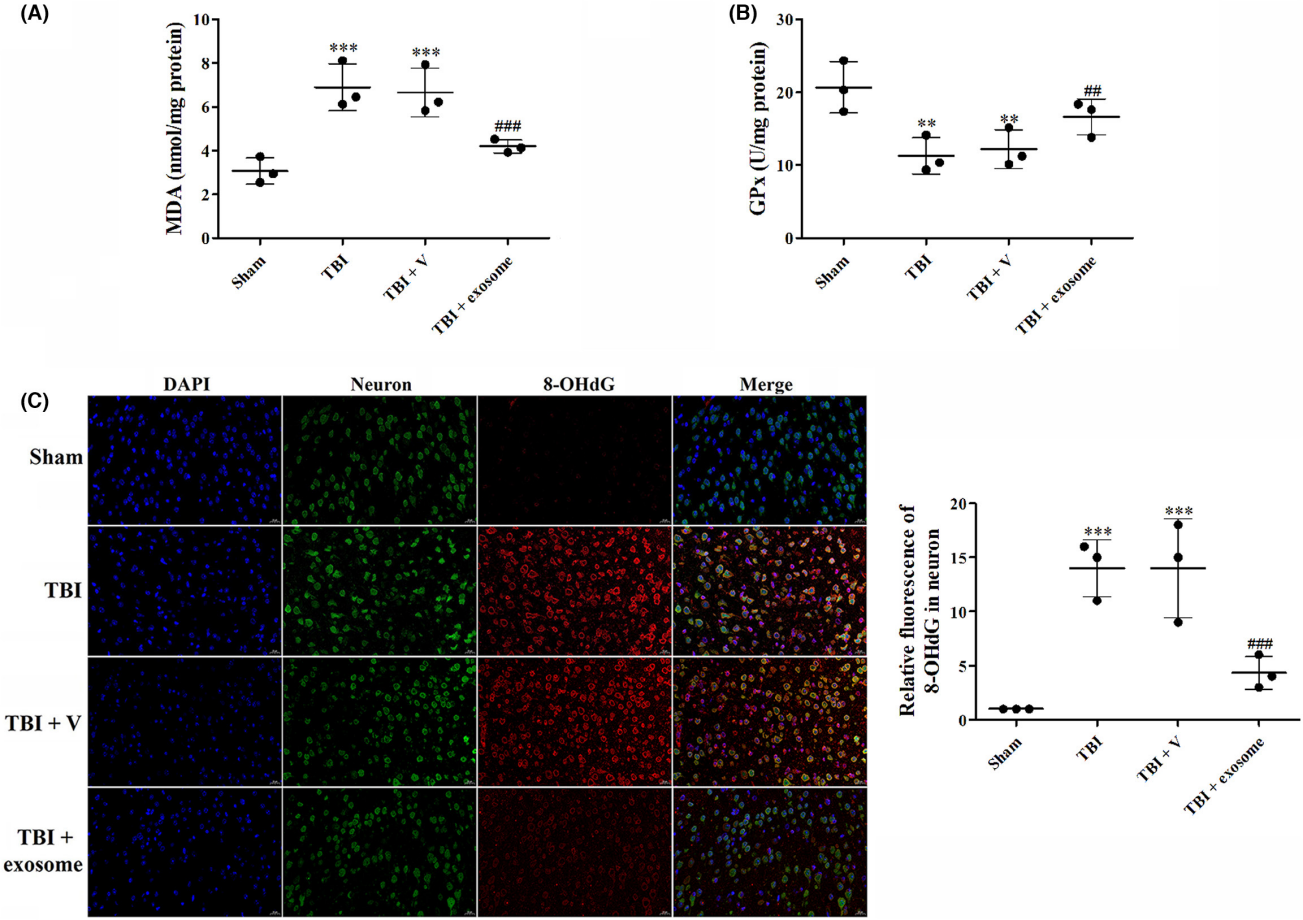
Exosome inhibited TBI‐induced oxidative damage. (A, B) MDA levels (A) and Gpx activity (B) was evaluated by ELISA. (C) Representative images of IF staining for 8‐OHdG in all groups. Data were presented as mean ± SD; **p < 0.01, ***p < 0.001 versus sham group; ^##^ *p* < 0.01, ^###^ *p* < 0.001 versus TBI + vehicle group. Scale bar: 20 μm.

Current studies have indicated that ferroptosis was a crucial mechanism causing neurological deficit of TBI, and inhibition of ferroptosis may improve long‐term outcomes of TBI. The characteristics of ferroptosis are the iron‐dependent lipid peroxidation (LPO) accumulation and aggravated oxidative damage. To confirm that ferroptosis was initiated after TBI and exosome could alleviate TBI‐induced ferroptosis, we first analyzed the LPO levels by measurement of MDA and GPx 1 day after TBI. We found that the levels of MDA were significantly up‐regulated after TBI, however, treatment of exosome down‐regulated the level of MDA (Figure 6A). GPx, in contrast, was down‐regulated after TBI. While the levels of GPx were reversed after exosome treatment (Figure 6B). IF staining of 8‐OHdG also showed TBI‐aggravated oxidative deoxyribonucleic acid (DNA) damage in the cortical neuron, but exosome treatment inhibited TBI‐induced oxidative damage (Figure 6C).

**FIGURE 7:**
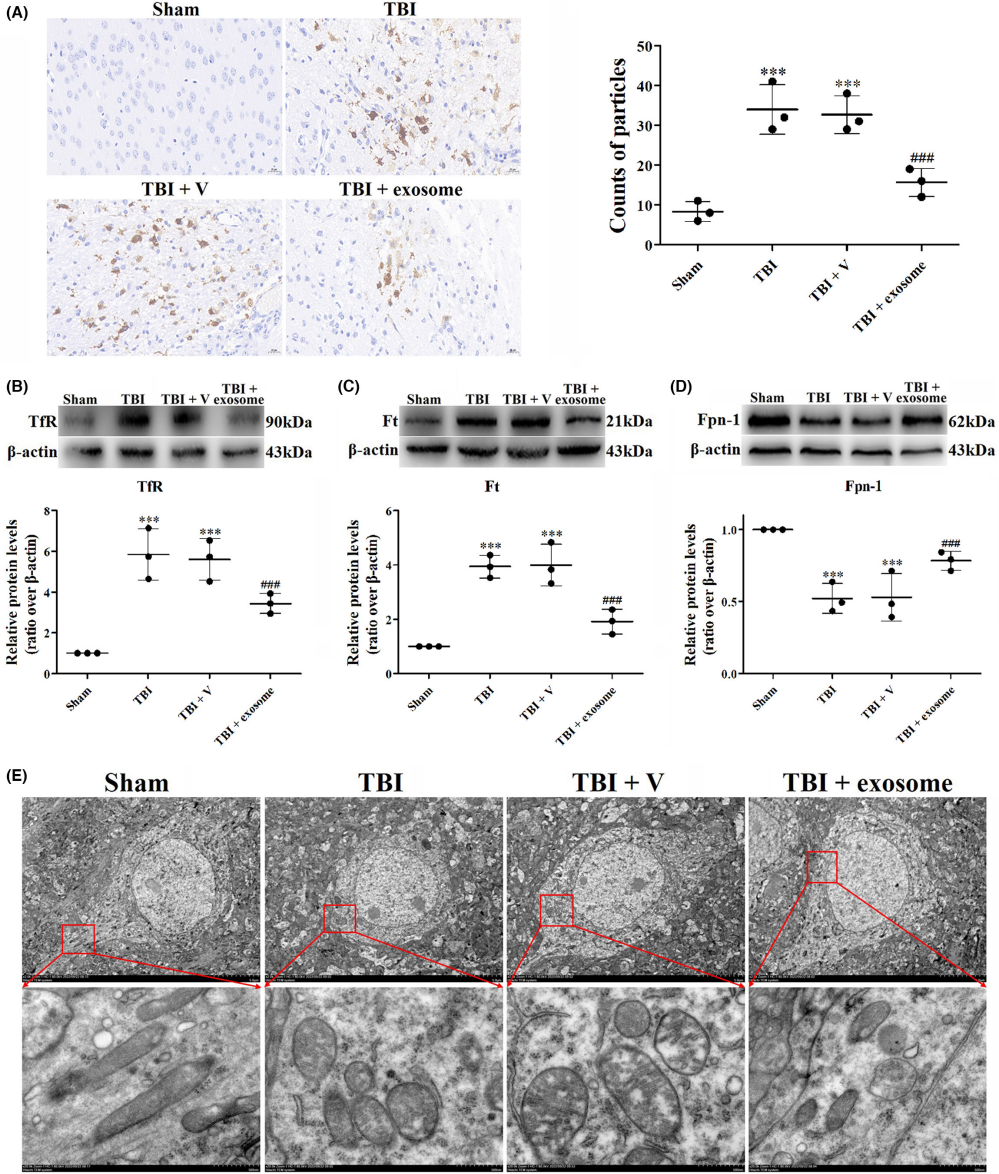
Exosome inhibited TBI‐induced iron accumulation and mitochondrial damage. (A) Perls' DAB staining was used to assay iron accumulation. (B–D) Western blot assay for the expression of TfR (B), Ft (C) and Fpn‐1 (D) in the ipsilateral cortex after TBI and exosome treatment. (E) TEM photomicrographs of mitochondria after TBI and exosome treatment. Data were presented as mean ± SD; ***p < 0.001 versus sham group; ^###^ *p* < 0.001 versus TBI + vehicle group. Scale bar: 20 μm. β‐Actin was used as a loading control.

Abnormal iron homeostasis is an important activator of ferroptosis and leads to a pathological state of CNS. We then investigated iron homeostasis after TBI. Iron homeostasis is closely mediated by TfR internalization for iron uptake, Ft for iron storage and Fpn‐1 for iron export in cells. Perls' DAB staining showed that cellular iron was accumulated after TBI, treatment with exosome obviously inhibited the iron levels at 1 day post‐TBI (Figure 7A). Besides, the expression of TfR and Ft was increased, and the expression of Fpn‐1 was decreased following TBI, suggesting that iron was transported to cells. Administration of exosome decreased the expression of TfR and Ft and increased the expression of Fpn‐1 compared with the TBI + vehicle group (Figure 7B–D), indicating that TBI‐induced accumulation of iron was reversed by exosome.

Next, we observed the cell morphology by TEM. Upon TBI, mitochondria became smaller with the mitochondrial ridges decreasing. In addition, the bilayer membrane density was also increased, which was considered as the symbol of ferroptosis (Figure 7E). However, exosome treatment restored the morphological changes of mitochondria (Figure 7E).

**FIGURE 8:**
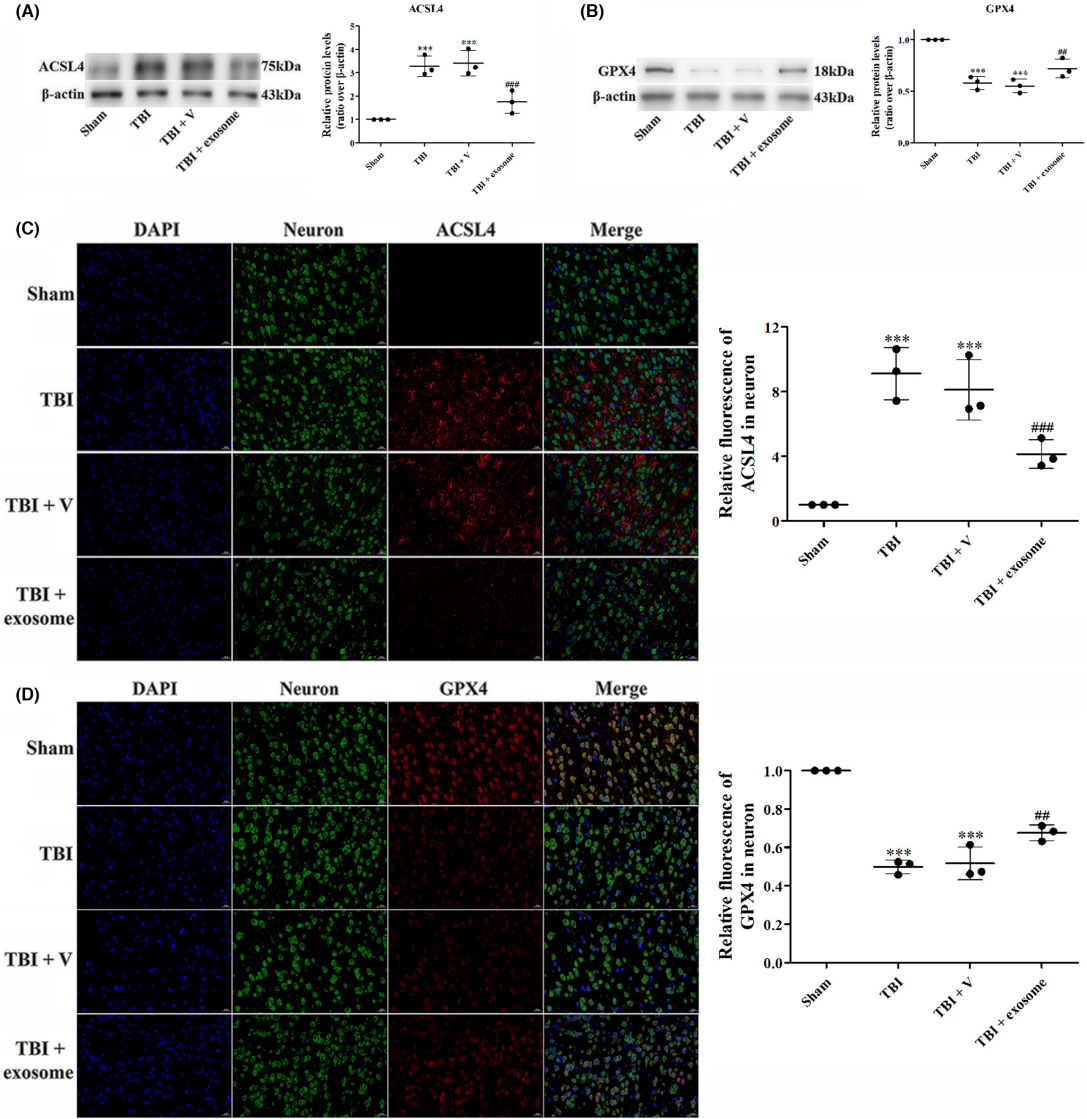
Exosome decreased the expression of ferroptosis‐related proteins. (A, B) Western blot assay for the expression of ACSL4 (A) and GPX4 (B) in the ipsilateral cortex after TBI and exosome treatment. (C, D) Representative images of IF staining for ACSL4 (C) and GPX4 (D) in all groups. Data were presented as mean ± SD; ***p < 0.001 versus sham group; ^##^ *p* < 0.01, ^###^ *p* < 0.001 versus TBI + vehicle group. Scale bar: 20 μm. β‐Actin was used as a loading control.

The expression of ferroptosis‐related protein was also analyzed after TBI. It has been shown that ACSL4 and GPX4 are the main targets in the regulation of ferroptosis. We then measured the expression of these proteins by Western blot and IF. Compared with sham group, the expression of ACSL4 were up‐regulated after TBI (Figure 8A,C), accompanied by a decreased expression of GPX4, suggesting that TBI‐activated ferroptosis. However, treatment of exosome suppressed TBI‐induced ferroptosis as evidenced by decreased expression of ACSL4 (Figure 8A,C) and increased expression of GPX4 (Figure 8B,D).

### 3.5 Exosome promoted mitophagy to inhibit brain injury after TBI

**FIGURE 9:**
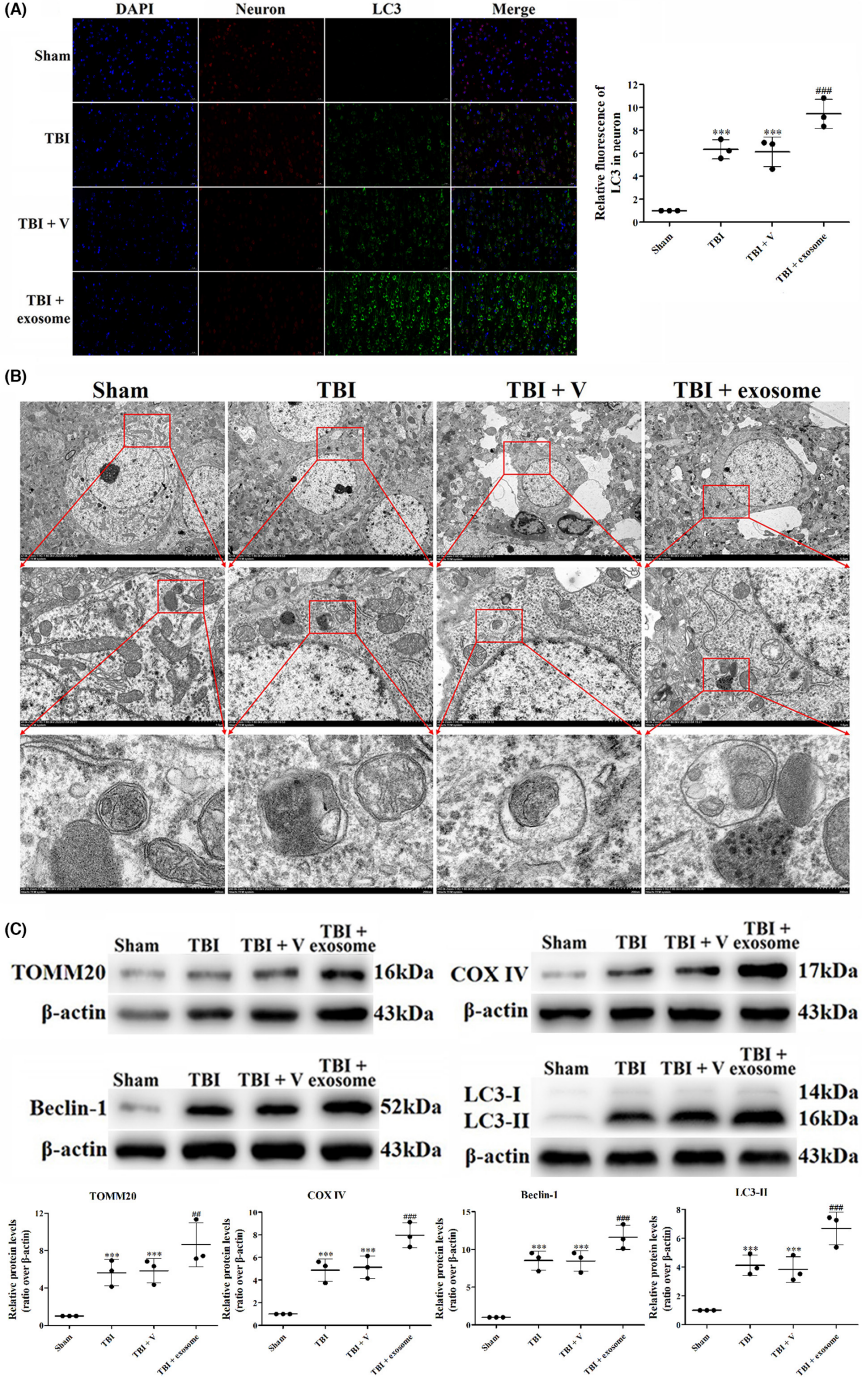
Exosome activated mitophagy after TBI. (A) Representative images of IF staining for LC3 in all groups. (B) TEM photomicrographs of autophagic vacuoles containing cellular material or organelle after TBI and exosome treatment. (C) Western blot assay for the expression of TOMM20, COX IV, Beclin‐1, and LC3 in the ipsilateral cortex after TBI and exosome treatment. Data were presented as mean ± SD; ***p < 0.001 versus sham group; ^##^ *p* < 0.01, ^###^ *p* < 0.001 versus TBI + vehicle group. Scale bar: 20 μm. β‐Actin was used as a loading control.

Previous studies have demonstrated that inhibition of mitophagy contributed to brain injury after TBI. Therefore, we wondered whether mitophagy was involved in the protective effects of exosome against TBI. We firstly used the IF staining of LC3, results of the fluorescence microscopy revealed that the TBI groups exhibited a significant increase in the number of neurons with LC3 compared with the sham groups, suggesting the formation of autophagosome (Figure 9A). In addition, the LC3‐positive neurons were further increased after exosome treatment (Figure 9A). The ability of exosome to induce autophagy was confirmed by TEM. In respond to TBI, the autophagic vacuoles containing cellular material or organelle were observed in the cytoplasm. Upon treatment of exosome, the number of autophagic vacuoles was up‐regulated (Figure 9B). To further verify that mitophagy was induced by exosome, we analyzed the expression of mitophagy‐related proteins, such as TOMM20, COX IV, Beclin‐1 and LC3. We found that TBI increased the expression of TOMM20, COX IV, Beclin‐1, and LC3‐II (Figure 9C), suggesting the activation of mitophagy after TBI. Moreover, the TBI‐induced mitophagy was enhanced by exosome, as proven by further increased expression of TOMM20, COX IV, Beclin‐1, and LC3‐II (Figure 9C). These data illustrated that exosome could promote TBI‐induced mitophagy.

**FIGURE 10:**
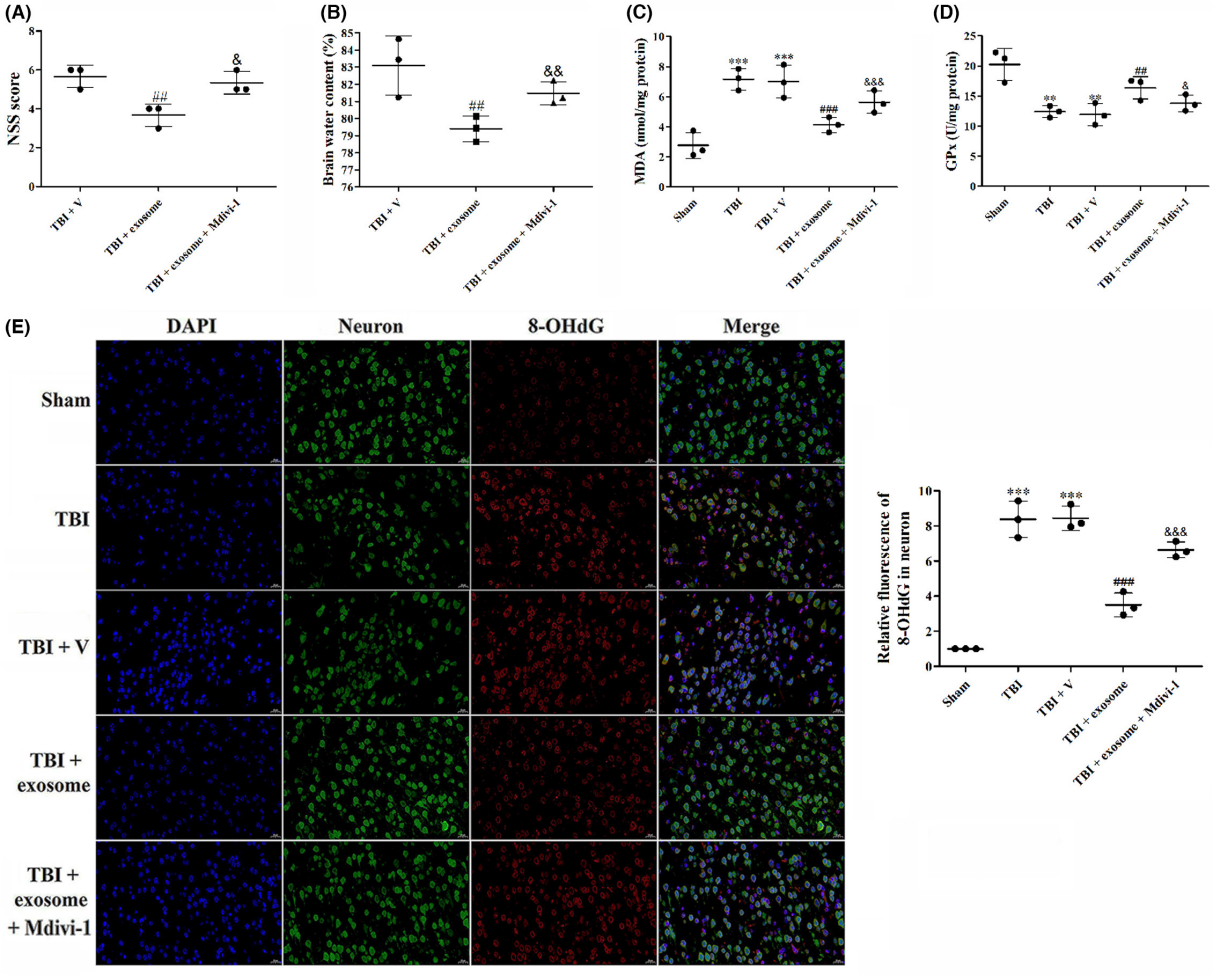
Suppression of mitophagy reversed the inhibitory effects of exosome on neurological deficit, brain edema, and oxidative dagame. (A) NSS was evaluated at 1 day after TBI, exosome treatment and suppression of mitophagy. (B) Brain water content was examined at 1 day after TBI, exosome treatment and suppression of mitophagy. (C, D) MDA levels (C) and Gpx activity (D) was evaluated by ELISA in all groups. (E) Representative images of IF staining for 8‐OHdG in all groups. Data were presented as mean ± SD; **p < 0.01, ***p < 0.001 versus sham group; ^##^ *p* < 0.01, ^###^ *p* < 0.001 versus TBI + vehicle group. ^&^ *p* < 0.05, ^&&^ *p* < 0.01, ^&&&^ *p* < 0.001 versus TBI + exosome group. Scale bar: 20 μm.

**FIGURE 11:**
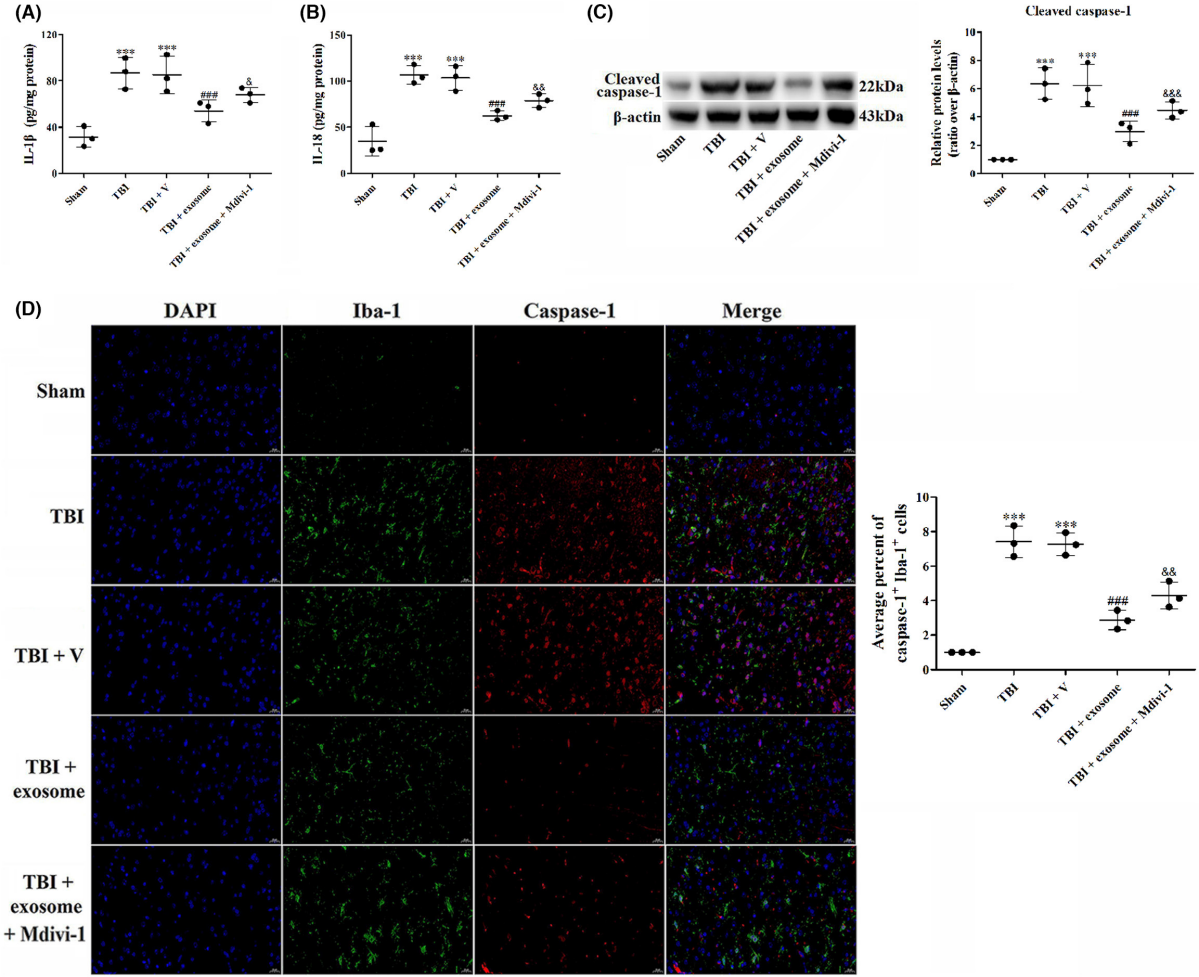
Suppression of mitophagy reversed the inhibitory effects of exosome on inflammation and pyroptosis. (A, B) Quantification of IL‐1β (A) and IL‐18 (B) at 1 day after TBI, exosome treatment, and inhibition of mitophagy. (C) Western blot assay for the expression of cleaved caspase‐1 after TBI, exosome treatment, and suppression of mitophagy. (D) Representative images of IF staining for cleaved caspase‐1 and Iba‐1 in all groups. Data were presented as mean ± SD; ***p < 0.001 versus sham group; ^###^ *p* < 0.001 versus TBI + vehicle group. ^&^ *p* < 0.05, ^&&^ *p* < 0.01, ^&&&^ *p* < 0.001 versus TBI + exosome group. Scale bar: 20 μm. β‐Actin was used as a loading control.

**FIGURE 12:**
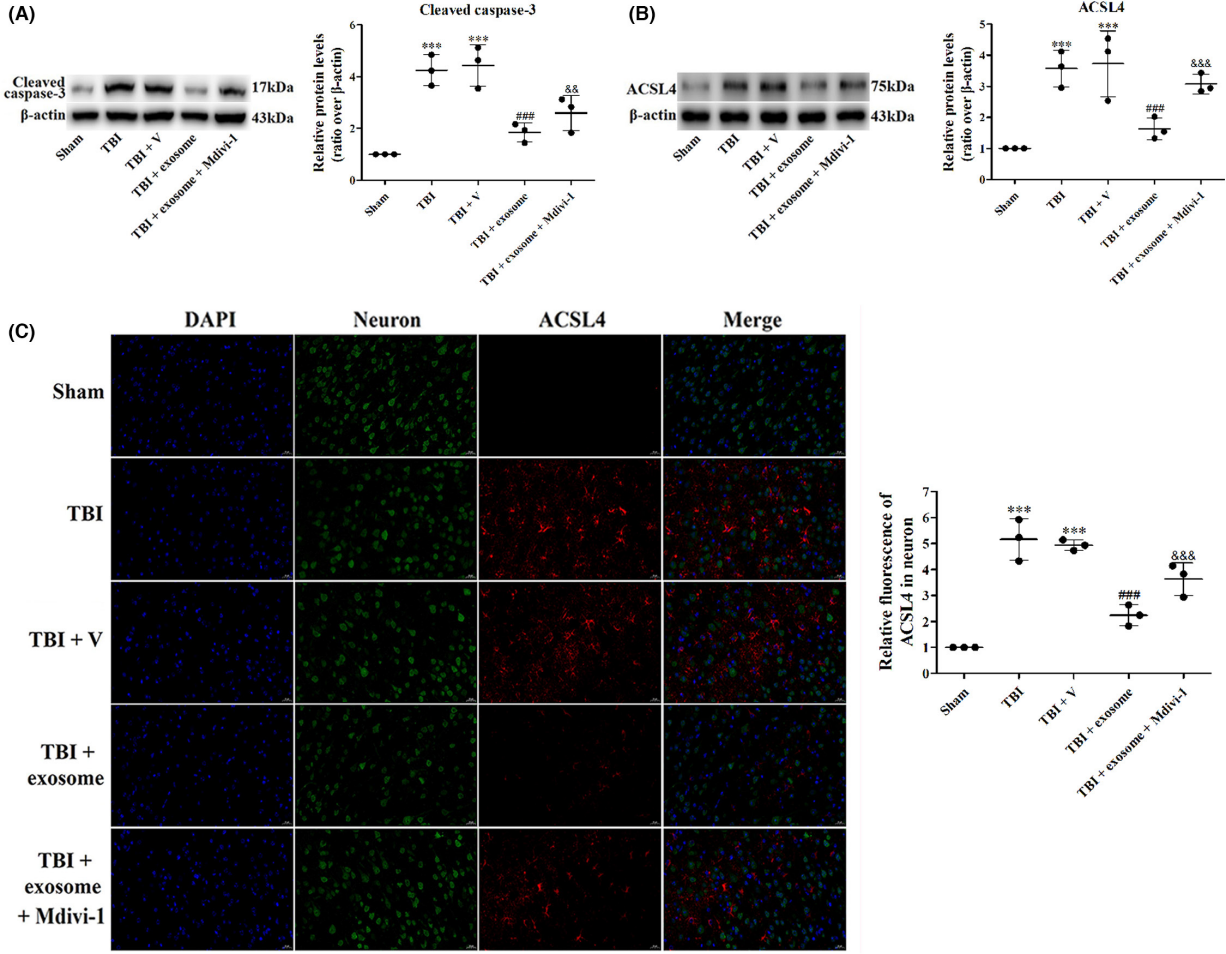
Suppression of mitophagy reversed the inhibitory effects of exosome on apoptosis and ferroptosis. (A, B) Western blot assay for the expression of cleaved caspase‐3 (A) and ACSL4 (B) in the ipsilateral cortex 1 day after TBI, exosome treatment, and suppression of mitophagy. (C) Representative images of IF staining for ACSL4 in all groups. Data were presented as mean ± SD; ***p < 0.001 versus sham group; ^###^ *p* < 0.001 versus TBI + vehicle group. ^&&^ *p* < 0.01, ^&&&^ *p* < 0.001 versus TBI + exosome group. Scale bar: 20 μm. β‐Actin was used as a loading control.

To determine whether the neuroprotective effects of exosome in TBI were abated when mitophagy was blocked, we used a mitophagy inhibitor mitochondrial division inhibitor‐1 (Mdivi‐1) and studied the neurologic function, brain water content, oxidative damage, inflammation, pyroptosis, apoptosis, and ferroptosis. As shown in Figure 11A,B, there was significant difference of the neurologic function and brain water content between the exosome‐treated and exosome + Mdivi‐1‐treated groups. Moreover, Mdivi‐1 partly reversed the inhibitory effects of exosome on oxidative damage (Figure 10C–E), inflammatory cytokines (Figure 11A,B), pyroptosis (Figure 11C,D), apoptosis (Figure 12A), and ferroptosis (Figure 12B,C) in TBI, suggesting that the mitophagy was partly involved in the neuroprotection of exosome.

### 3.6 Exosome provided neuroprotection in TBI via PINK1/Parkin pathway‐mediated mitophagy

**FIGURE 13:**
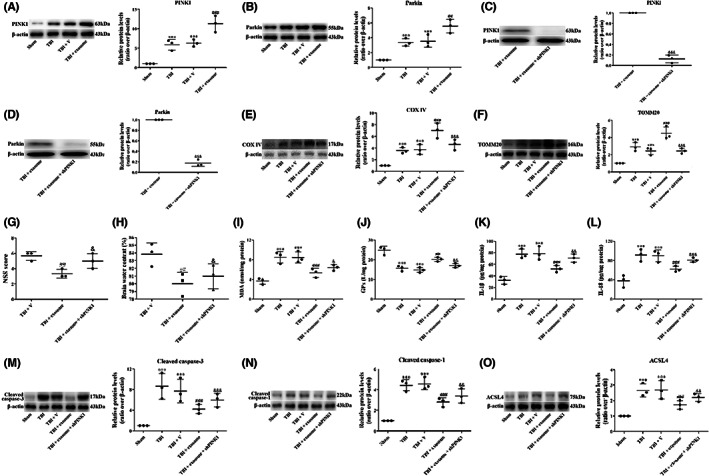
Exosome activated the PINK1/Parkin pathway‐mediated mitophagy. (A, B) Western blot assay for the expression of PINK1 (A) and Parkin (B) in the ipsilateral cortex 1 day after TBI and exosome treatment. (C, D) When PINK1 was knockdown, Western blot assay for the expression of PINK1 (C) and Parkin (D) was analyzed. (E, F) Western blot assay for the expression of COX IV (E) and TOMM20 (F) in the ipsilateral cortex 1 day after TBI, exosome treatment and knockdown of PINK1. (G) NSS was evaluated at 1 day after TBI, exosome treatment and knockdown of PINK1. (H) Brain water content was examined at 1 day after TBI, exosome treatment and knockdown of PINK1. (I, J) MDA levels (I) and Gpx activity (J) was evaluated by ELISA in all groups. (K, L) Quantification of IL‐1β (K) and IL‐18 (L) at 1 day in all groups. (M, N, O) Western blot assay for the expression of cleaved caspase‐3 (M), cleaved caspase‐1 (N) and ACSL4 (O) in the ipsilateral cortex 1 day after TBI, exosome treatment and knockdown of PINK1. Data were presented as mean ± SD; ***p < 0.001 versus sham group; ^##^ *p* < 0.01, ^###^ *p* < 0.001 versus TBI + vehicle group. ^&^ *p* < 0.05, ^&&^ *p* < 0.01, ^&&&^ *p* < 0.001 versus TBI + exosome group. β‐Actin was used as a loading control.

PINK1/Parkin pathway has been demonstrated to play a key role in mitophagy and brain injury after TBI. Thus, we analyzed the involvement of PINK1/Parkin pathway in the neuroprotective effects of exosome. The results showed that the expression of PINK1 and Parkin was significantly increased after TBI, and treatment of exosome further increased PINK1 and Parkin expression (Figure 13A,B). The effects of PINK1/Parkin pathway on mitophagy and brain damage were then studied by knockdown of PINK1. The expression of PINK1 and Parkin was significantly decreased by knockdown of PINK1 (Figure 13C,D). Moreover, knockdown of PINK1 reversed the effects of exosome on mitophagy (Figure 13E,F), neurological deficit (Figure 13G), brain edema (Figure 13H), oxidative damage (Figure 13I,J), inflammatory cytokines (Figure 13K,L), apoptosis (Figure 13M), pyroptosis (Figure 13N), and ferroptosis (Figure 13O), indicating that exosome provided neuroprotection in TBI via PINK1/Parkin pathway‐mediated mitophagy.

### 3.7 Exosome protected primary cultured neurons from TBI

**FIGURE 14:**
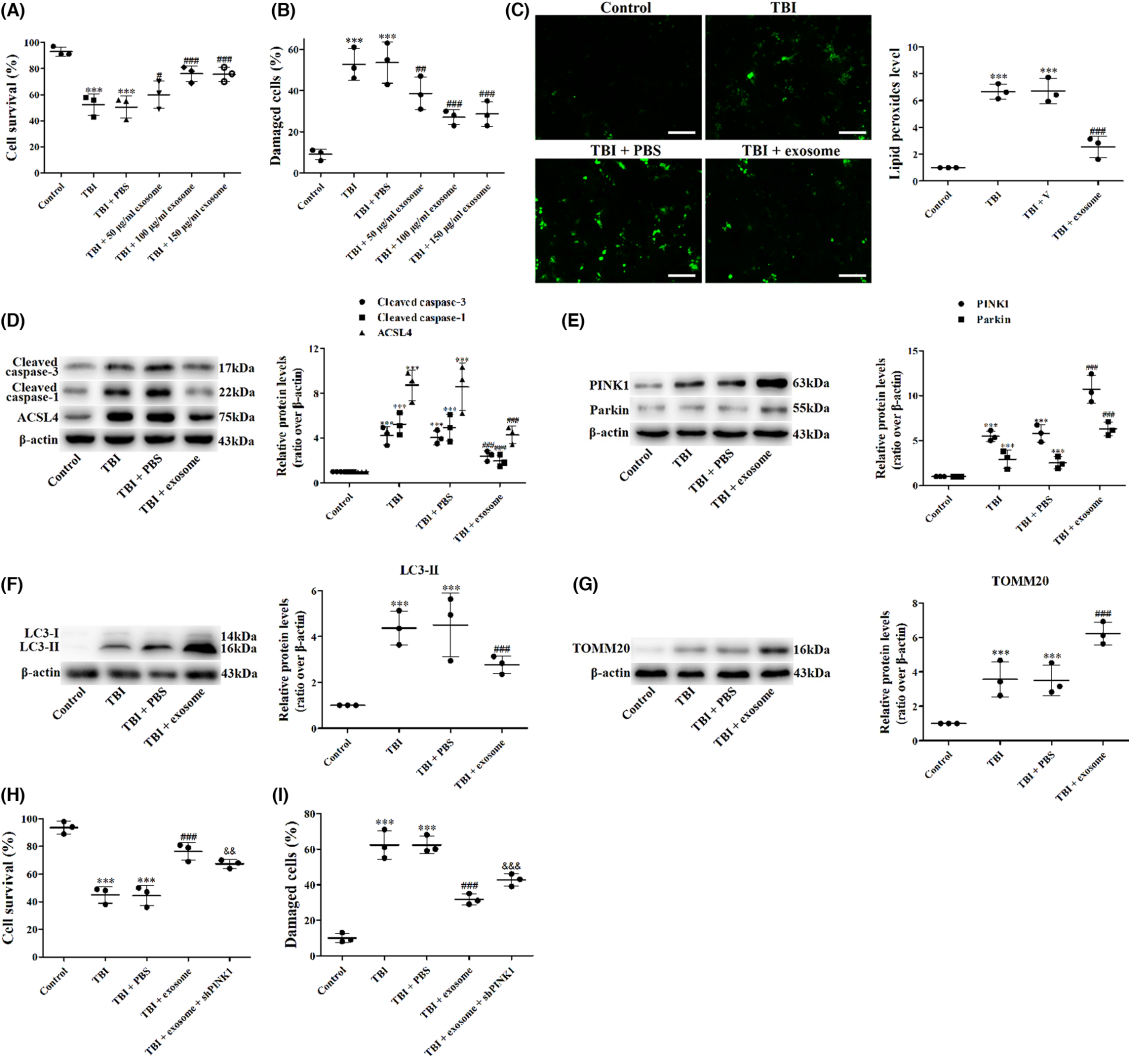
Exosome protected primary cultured neurons from TBI. (A, B) Primary cortical neurons were subjected to scratch injury and then treated with 50 μg/mL, 100 μg/mL, and 150 μg/mL exosome or PBS for 1 day. The TB staining (A) and LDH release assay (B) were used to evaluate cell viability. (C) BODIPY staining of lipid peroxides in primary cultured cells at 1 day after TBI and exosome treatment. (D) Western blot assay for the expression of cleaved caspase‐3, cleaved caspase‐1, and ACSL4 in primary cultured cells at 1 day after TBI and exosome treatment. (E) Western blot assay for the expression of PINK1 and Parkin in primary cultured cells at 1 day after TBI and exosome treatment. (F, G) Western blot assay for the expression of LC3 (F) and TOMM20 (G) in primary cultured cells at 1 day after TBI and exosome treatment. (H, I) The TB staining (H) and LDH release assay (I) were used to evaluate cell viability in primary cultured cells at 1 day after TBI, exosome treatment and knockdown of PINK1. Data were presented as mean ± SD; ***p < 0.001 versus control group; ^#^ *p* < 0.05, ^##^ *p* < 0.01, ^###^ *p* < 0.001 versus TBI + PBS group; ^&&^ *p* < 0.01, ^&&&^ *p* < 0.001 versus TBI + exosome group. Scale bar: 50 μm. β‐Actin was used as a loading control.

The neuroprotective effects of exosome in TBI were also confirmed in primary‐cultured neurons. TB staining and LDH release assay were firstly conducted in neurons treated with exosome. In TB staining, treatment of exosome significantly increased the percentage of viable cells (Figure 14A). Similar results were found in LDH release assay (Figure 14B). These results suggested that exosome provided neuroprotection after TBI in vitro and indicated that 100 μg/mL of exosome exhibited the best effect, which we used in the following in vitro experiments.

Then, to understand the effects of exosome on neuron lipid peroxides level, we used BODIPY staining. Figure 14C showed that compared with the control neurons, the level of lipid peroxides in the damaged neurons was significantly increased. When neurons were treated with exosome, the level of lipid peroxides was decreased (Figure 14C).

We next analyzed the expression of cleaved caspase‐3, cleaved caspase‐1, ACSL4, PINK1, Parkin, LC3, and TOMM20 by western blot. We found that compared with the control neurons, the expression of cleaved caspase‐3, cleaved caspase‐1, ACSL4, PINK1, Parkin, LC3‐II, and TOMM20 was increased in the damaged neurons (Figure 14D–G). Exosome treatment reduced the expression of cleaved caspase‐3, cleaved caspase‐1, and ACSL4 but further increased the expression of PINK1, Parkin, LC3‐II, and TOMM20 (Figure 14D–G). These results demonstrated that exosome increased cell viability, suppressed lipid peroxides, decreased apoptosis, pyroptosis, and ferroptosis and activated the mitophagy after TBI in vitro. However, when PINK1 was knockdown, the protection of exosome on neuronal cell death was partly attenuated (Figure 14H,I), demonstrating that exosome provided neuroprotective effects in primary cultured neurons partly through the PINK1/Parkin pathway‐mediated mitophagy after TBI.

## 4 DISCUSSION

To the best of our knowledge, this is the first study detailly examining the effects of HucMSC‐derived exosome on PCD in TBI. The main findings were as follows: (1) HucMSC‐derived exosome improved neurological function, decreased cerebral edema, and attenuated lesion volume after TBI. (2) HucMSC‐derived exosome suppressed TBI‐induced cell death, apoptosis, pyroptosis, and ferroptosis. (3) HucMSC‐derived exosome activated the PINK1/Parkin‐mediated mitophagy after TBI. (4) Inhibition of PINK1/Parkin‐mediated mitophagy reversed the neuroprotection of HucMSC‐derived exosome in TBI.

Recent studies have recognized the importance of pyroptosis in CNS injuries. It has been shown that pyroptosis played an important role in the progression of neonatal cerebral ischemia–reperfusion (I/R) injury.^28^ Additionally, Hu et al. suggested that mesenchymal stem cell (MSC)‐derived exosome protected against oxygen–glucose deprivation/reperfusion (OGD/R)‐induced brain damage by suppressing pyroptosis in microglia.^19^ Pyroptosis is a type of inflammatory PCD that is characterized by the formation of pores in cell membrane, cell lysis, and release of pro‐inflammatory cytokines.^27^ Pyroptosis is primarily induced by inflammasomes and performed by the gasdermin (GSDM) protein family such as GSDMD and caspases such as caspase‐1.^29^ Inflammasomes are multimolecular complexes containing pattern‐recognition receptors (PRR), apoptosis‐associated speck‐like protein containing CARD (ASC) and NLRP3.^30^ Growing evidence has shown that NLRP3 inflammasome‐mediated pyroptosis was involved in the pathogenesis of TBI. Yang et al. revealed that Gastrodin suppressed NLRP3 inflammasome signaling pathway, and therefore inhibited pyroptosis, reduced brain tissue injury, and improved functional recovery of injury nerve in TBI rats.^31^ Moreover, Yuan et al. found that interference of hypoxia‐inducible factor‐1α (HIF‐1α) decreased the expressions of NLRP3, GSDMD‐N and cleaved caspase‐1 in the injured cortex after TBI, suggesting that HIF‐1α aggravated NLRP3 inflammasome‐mediated pyroptosis in TBI.^32^ In the present study, we investigated whether exosome inhibited pyroptosis in TBI. Similar to these studies, we found that exosome significantly inhibited the release of inflammatory factor such as IL‐1β, IL‐6, IL‐18, and downregulated the expression levels of the key proteins associated with pyroptosis such as NLRP3, GSDMD‐N and cleaved caspase‐1 in TBI. These findings suggested that the protective effects of exosome against TBI was associated with the inhibition of pyroptosis.

Ferroptosis is a recently discovered form of non‐apoptotic PCD.^33^ Biologically, the characteristics of ferroptosis are the iron‐dependent LPO accumulation.^34^ Ferroptosis plays a key role in TBI and is closely related to oxidative stress, immunity, and chronic injuries. The inhibitors against ferroptosis effectively improve iron homeostasis, lipid metabolism, redox stabilization, neuronal remodeling, and functional recovery after trauma.^35^ For example, Wang et al. indicated that repetitive mild traumatic brain injury (rmTBI) caused time‐dependent alterations in ferroptosis‐related biomarker levels. However, treatment of mesenchymal stromal cells (MSCs) markedly decreased the rmTBI‐mediated ferroptosis.^36^ Moreover, Cheng et al. reported that TBI induced ferroptosis as proved by increased expression of TfR1, decreased levels of MDA, iron homeostatic imbalance and generation of LPO, while treatment of ferristatin II could reduce the expression of TfR1, suppress iron homeostatic imbalance, increase MDA level and attenuate LPO following TBI, suggesting that ferristatin II provided neuroprotection against TBI by inhibiting ferroptosis.^37^ In the present study, we also examined the effects of exosome on TBI‐induced LPO, oxidative damage, iron accumulation, and mitochondrial damage. It has been shown that MDA, which indicated lipid peroxidation traumatized tissue, began to up‐regulated immediately after TBI, and remained at the highest level 1 day after injury.^38^ On the contrary, the antioxidant enzymes such as GPx were used to eliminate metabolites produced by free radicals, transforming peroxides into innocuous substances, and their activities underwent a descent and minimum 1 day post‐injury.^39^ Consistent with these results, we found that TBI increased the level of MDA and decreased the level of GPx. After being treated with exosome, the MDA level down‐regulated, the GPx level up‐regulated and the oxidative‐antioxidant system rebalanced. Furthermore, 8‐OHdG is one of the predominant forms of free radical‐induced oxidative lesions, which is considered as a critical biomarker of oxidative damage.^40^ Perls' DAB is a staining procedure specific for Fe. It is widely applicable in pathophysiological CNS to detect Fe in frozen tissue sections. Perls' DAB staining is simple to operate, stable in performance and clear in color.^41^ Our data suggested that exosome could suppress TBI‐induced oxidative DNA damage and iron accumulation as evidenced by 8‐OHdG staining and Perls' DAB staining, respectively. Further studies found that exosome reduced iron accumulation by decreasing iron uptake, storage and increasing iron export, thus improving iron metabolism disturbances.

Morphologically, ferroptosis is mainly manifested by mitochondrion condensation or swelling, increased membrane density, decreased crista and ruptured outer membrane.^42^ Mechanically, ferroptosis can be activated by ACSL4 and inhibited by GPX4.^43^ Both ACSL4 and GPX4 are key regulators for protecting cells from LPO, which hinder the reduction in LPO and lead to ROS generation through the Fenton reaction‐dependent oxidation of lipids by Fe^2+^.^44^ In our study, we then examined the mitochondrial morphology by TEM and the expression of ACSL4, GPX4 by western blot and IF. In agreement with the former study, we found that the mitochondrial morphology was changed after TBI. However, these changes were reversed by exosome. In addition, the expression of ACSL4 were increased after TBI, accompanied by a decreased expression of GPX4, suggesting that TBI activated ferroptosis. Treatment of exosome suppressed TBI‐induced ferroptosis by down‐regulating the expression of ACSL4 and up‐regulating the expression of GPX4.

Mitophagy is the selective autophagy of mitochondria.^45^ It is an evolutionarily conserved mechanism that removes undesired and damaged mitochondria to adjust their number and maintain a balanced energy metabolism.^46^ Regulation of mitophagy also attenuated brain injury induced by TBI. It has been shown that TBI induced mitochondrial fission (represented by Drp1), mtDNA concentration down‐regulation and mitophagy activation. Inhibition of Drp1 by Mdivi‐1 suppressed mitophagy, alleviated BBB disruption, and cell apoptosis after TBI.^47^ Moreover, Lin et al. demonstrated that melatonin could attenuate TBI‐induced inflammation and activate mitophagy through the mTOR pathway, while 3‐methyladenine (3‐MA) reversed this effect by inhibiting mitophagy.^48^ We then wondered whether mitophagy was activated by exosome. In accordance with previous results, our data showed that TBI significantly up‐regulated the expression of mitophagy‐related proteins such as TOMM20, COX IV, Beclin‐1, and LC3‐II, exosome treatment further increased the expression of these proteins. In addition, both IF and TEM confirmed that mitophagy was activated after TBI and further promoted by exosome.

Recently, the interplay between mitophagy and different types of PCD, such as apoptosis, pyroptosis and ferroptosis has been proposed. In a rat subarachnoid hemorrhage (SAH) model, low serum triiodothyronine treatment reduced neuronal apoptosis and promoted mitophagy following SAH, inhibition of mitophagy by PINK1‐siRNA reversed this preventative effect on apoptosis.^49^ Moreover, Han et al. indicated that Quercetin decreased pyroptosis‐related proteins, including NLRP3, cleaved caspase‐1, GSDMD‐N, and cleaved IL‐1β through promoting mitophagy, thus preventing neuronal injury.^50^ Consist with these results, the present study showed that inhibition of mitophagy by Mdivi‐1 reversed the inhibitory effects of exosome on TBI‐induced cell death, apoptosis, pyroptosis, and ferroptosis, indicating that exosome prevented TBI‐induced PCD by enhancing mitophagy.

We further examined the potential activators of mitophagy in our system. The PINK1/Parkin‐mediated ubiquitin pathway is the main pathway that drives mitophagy.^51^ In Alzheimer's disease, Aβ1‐40 induced pericyte mitophagy through the PINK1/Parkin pathway.^52^ Under physiological conditions, PINK1 is transported to the inner mitochondrial membrane (IMM) and degraded by presenilin‐associated rhomboid‐like protease (PARL).^53^ However, in damaged mitochondria, the degradation of PINK1 is inhibited to maintain PINK1 present on the outer mitochondrial membrane (OMM).^54^ PINK1 accumulation on the OMM further mediates the phosphorylation of ubiquitin (Ub) at Ser65 and the phosphorylation of Ser65 in the ubiquitin‐like (UBL) domain of Parkin.^55^, ^56^ Then, phosphorylated Ub and Parkin promote mitophagosome formation by binding to LC3. Eventually, mitophagosome fuses with lysosome to form mitolysosome and lead to the elimination of damaged mitochondria.^51^ In our study, we found that the expression of PINK1 and Parkin was increased after TBI, treatment of exosome further increased the expression of PINK1 and Parkin. Interestingly, we found that knockdown of PINK1 partly inhibited mitophagy and reversed the neuroprotection of exosome, suggesting that there might be other pathways involved in the activation of mitophagy by exosome. This was an interesting aspect for us to explore in the future.

There were some limitations in our study. Firstly, exosome owns a variety of properties such as release of non‐coding RNAs. Thus, it should be clarified that whether the neuroprotection of exosome in TBI was contributed to other properties. Secondly, further studies were needed to investigate whether administration of exosome in different time courses might provide better neuroprotective effects against TBI.

## 5 CONCLUSION

Our study indicated that HucMSC‐derived exosome could provide neuroprotection against TBI by combating cell death, apoptosis, pyroptosis, and ferroptosis through the PINK1/Parkin‐mediated mitophagy. These results demonstrated that HucMSC‐derived exosome may be a promising target for the treatment of TBI.

## AUTHOR CONTRIBUTIONS

Li Zhang was responsible for the data collection, animal experiments, and manuscript writing. Yixing Lin was responsible for the design of the article. Wanshan Bai was responsible for the literature collection and manuscript review. Lean Sun was responsible for the data analysis. Mi Tian was responsible for the cell experiments and funding support. All authors read and approved the final manuscript.

## FUNDING INFORMATION

This work was supported by Grants from the National Natural Science Foundation of China (No. 82202392) from Mi Tian.

## CONFLICT OF INTEREST STATEMENT

The authors declare no conflict of interest.

## Supporting information


Appendix S1.

## Data Availability

The data used to support the findings of this study are available from the corresponding author upon request.
