# Supplementary material for: Human umbilical cord mesenchymal stem cell‐derived exosome suppresses programmed cell death in traumatic brain injury via PINK1/Parkin‐mediated mitophagy
Source: CNS Neurosci Ther. 2023 Mar 8;29(8):2236–58. doi: 10.1111/cns.14159 (PMC10352888; doi:10.1111/cns.14159)

Figure 2C

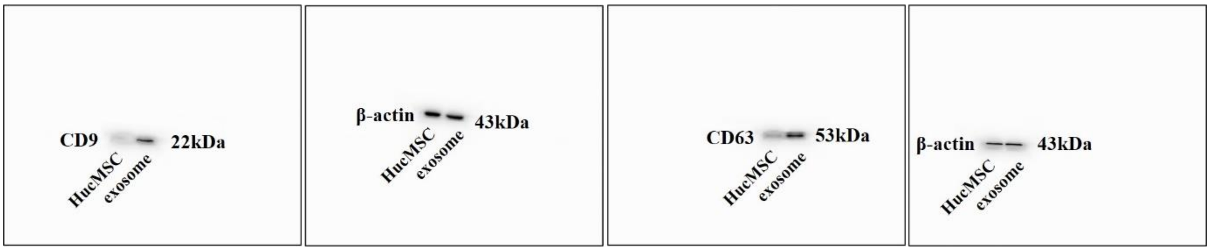

Figure 3C

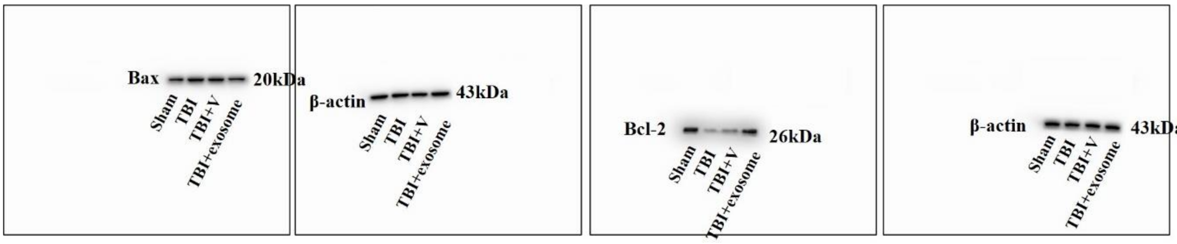

Figure 3D

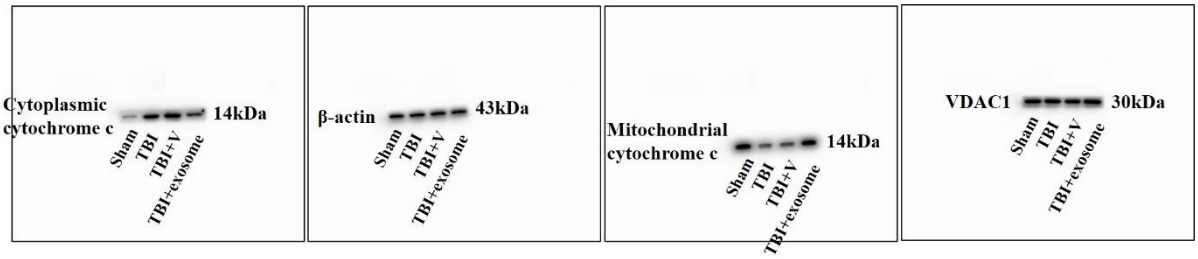

Figure 3E

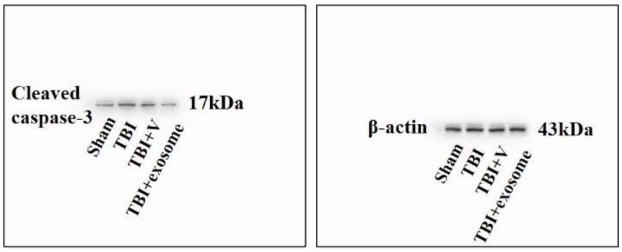

Figure 4B

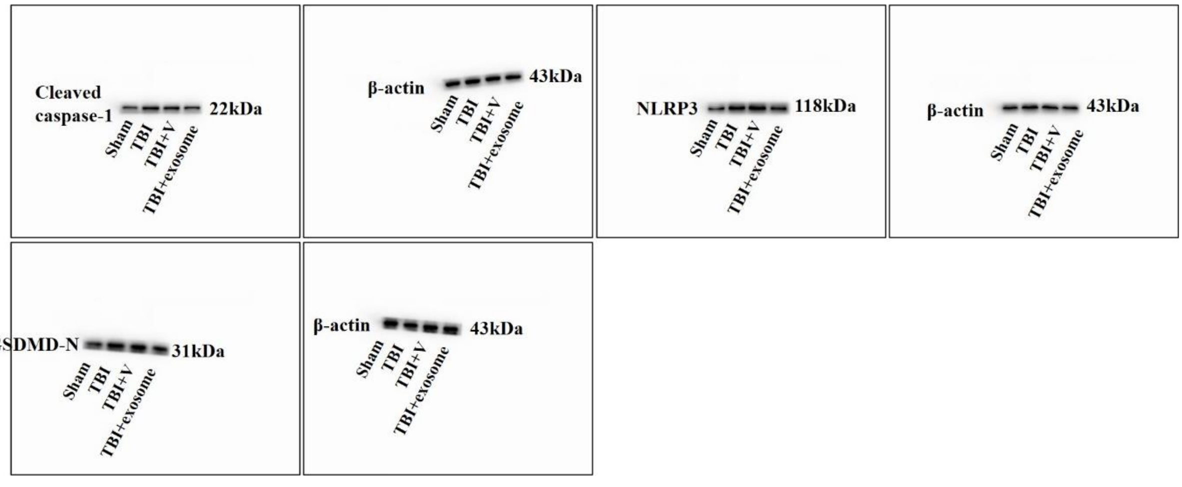

Figure 7B

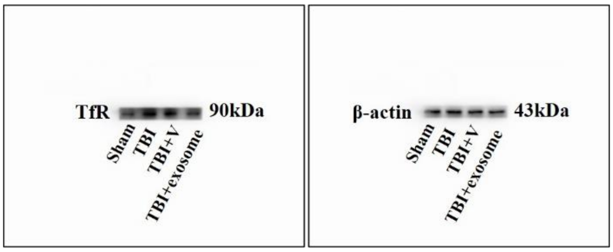

Figure 7C

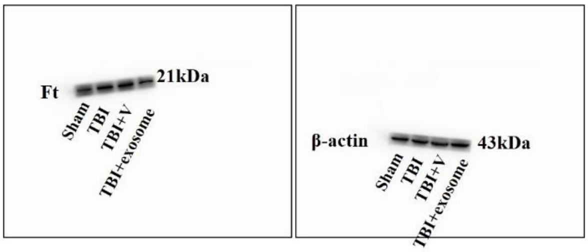

Figure 7D

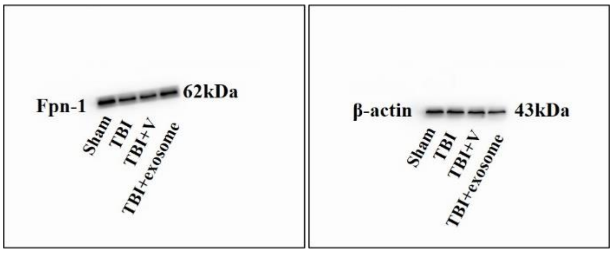

Figure 8A

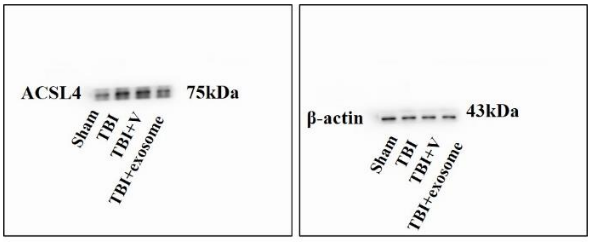

Figure 8B

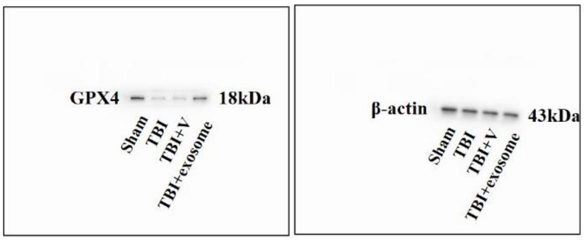

Figure 9C

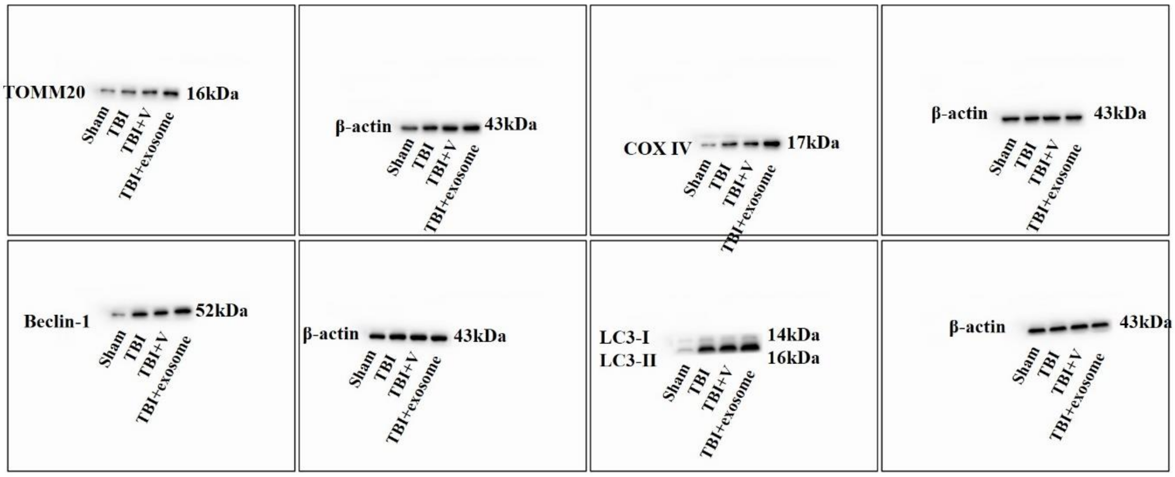

Figure 11C

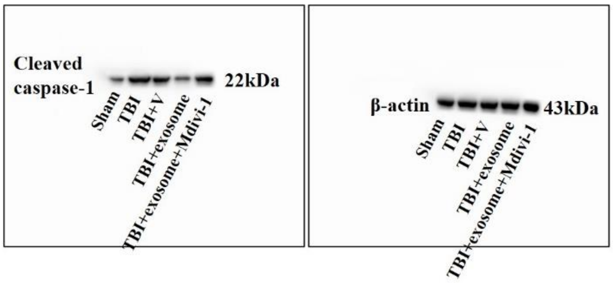

Figure 12A

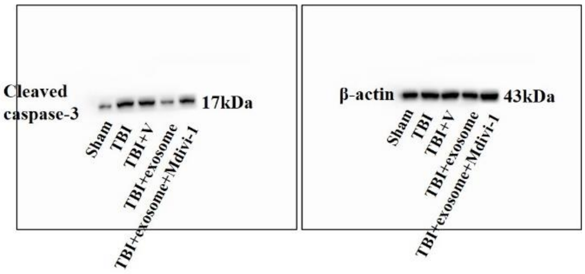

Figure 12B

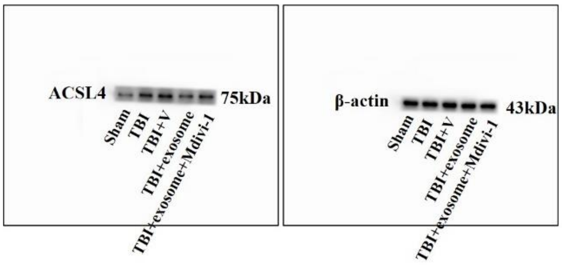

Figure 13A

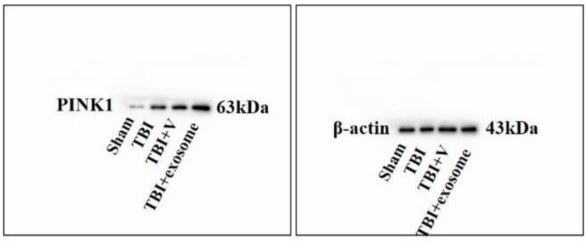

Figure 13B

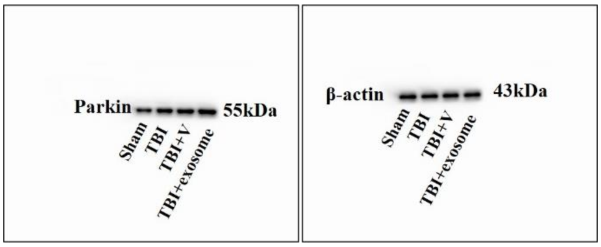

Figure 13C

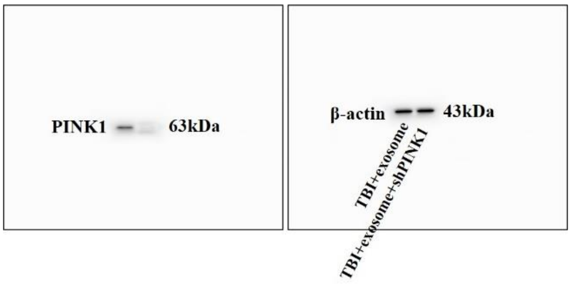

Figure 13D

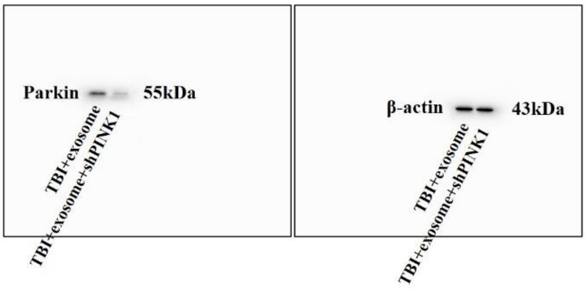

Figure 13E

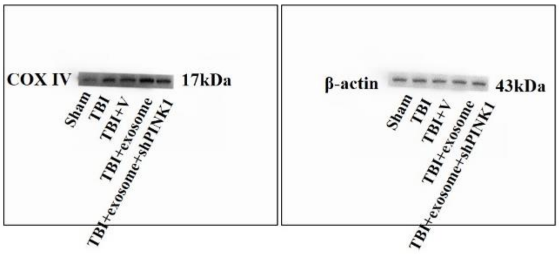

Figure 13F

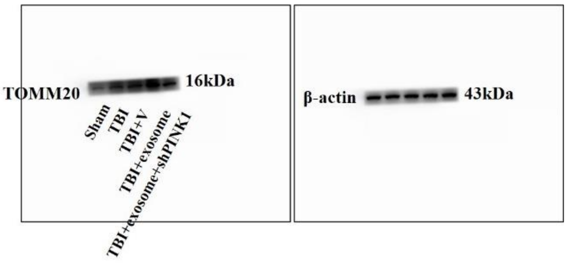

Figure 13M

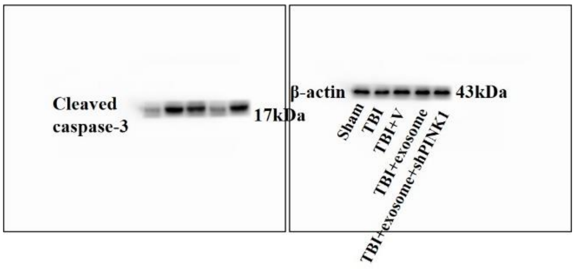

Figure 13N

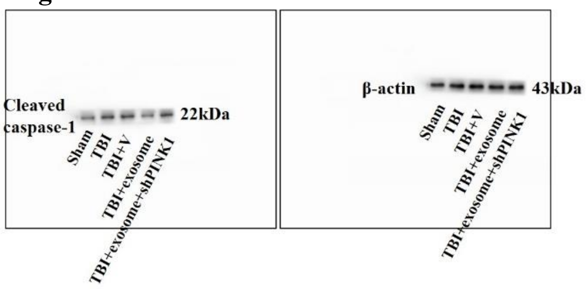

Figure 13O

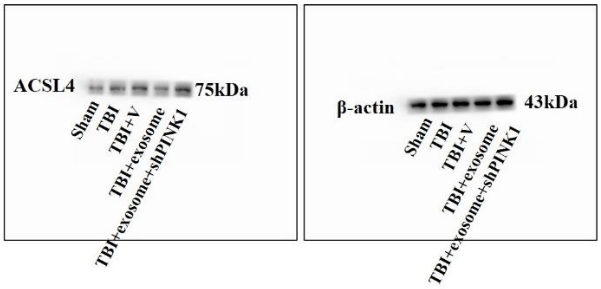

Figure 14D

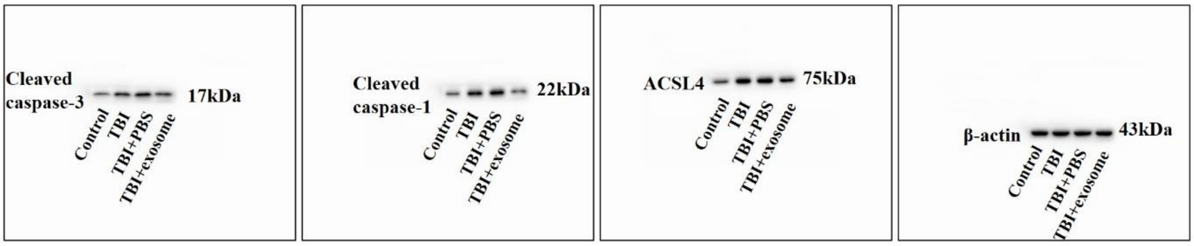

Figure 14E

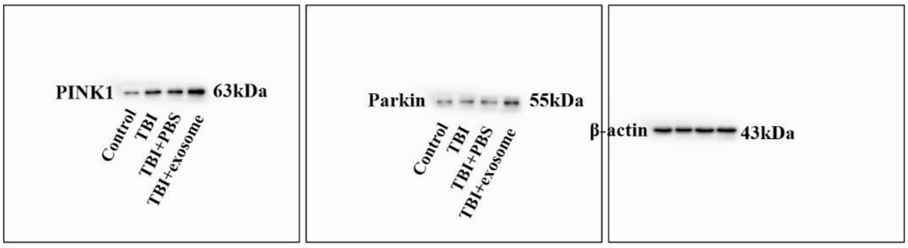

Figure 14F

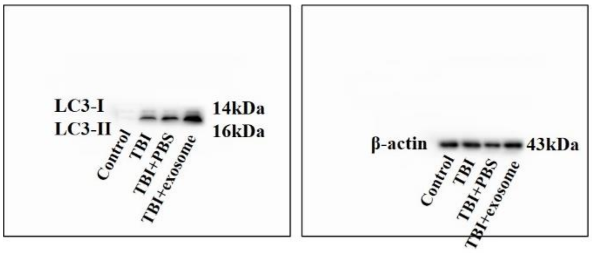

Figure 14G

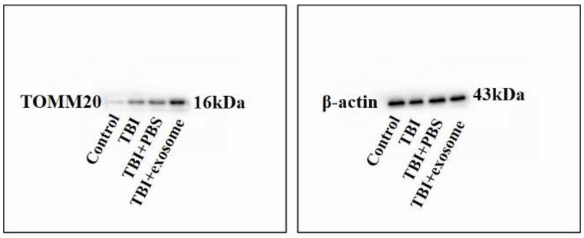

Supplement: Supplementary file 1 — Appendix S1. [file CNS-29-2236-s001.pdf]
